# Tri-explosophoric groups driven fused energetic heterocycles featuring superior energetic and safety performances outperforms HMX

**DOI:** 10.1038/s41467-022-33413-7

**Published:** 2022-09-28

**Authors:** Jie Li, Yubing Liu, Wenqi Ma, Teng Fei, Chunlin He, Siping Pang

**Affiliations:** 1 Experimental Center of Advanced Materials, School of Materials Science & Engineering, Beijing Institute of Technology, Beijing, 10081 China; 2 State Key Laboratory of Explosion Science and Technology, Beijing Institute of Technology, Beijing, 10081 China; 3 Yangtze Delta Region Academy of Beijing Institute of Technology, Jiaxing, 314019 China; 4 Chongqing Innovation Center, Beijing Institute of Technology, Chongqing, 401120 China

**Keywords:** Structure elucidation, Chemical safety, Synthetic chemistry methodology

## Abstract

The design and synthesis of novel energetic compounds with integrated properties of high density, high energy, good thermal stability and sensitivities is particularly challenging due to the inherent contradiction between energy and safety for energetic compounds. In this study, a novel structure of 4-amino-7,8-dinitropyrazolo-[5,1-*d*] [1,2,3,5]-tetrazine 2-oxide (BITE-101) is designed and synthesized in three steps. With the help of the complementary advantages of different explosophoric groups and diverse weak interactions, BITE-101 is superior to the benchmark explosive HMX in all respects, including higher density of 1.957 g·cm^−3^, highest decomposition temperature of 295 °C (onset) among CHON-based high explosives to date and superior detonation velocity and pressure (*D*: 9314 m·s^−1^, *P*: 39.3 GPa), impact and friction sensitivities (*IS*: 18 J, *FS*: 128 N), thereby showing great potential for practical application as replacement for HMX, the most powerful military explosive in current use.

## Abstract (web summary)

The design of high energy density materials (HEDM) with good detonation performance but which are also safe to handle is challenging. Here, the authors synthesize a PTX analogue and incorporate explosophoric groups to obtain an HEDM with improved detonation performance but low impact and friction sensitivity.

## Introduction

**Fig. 1:**
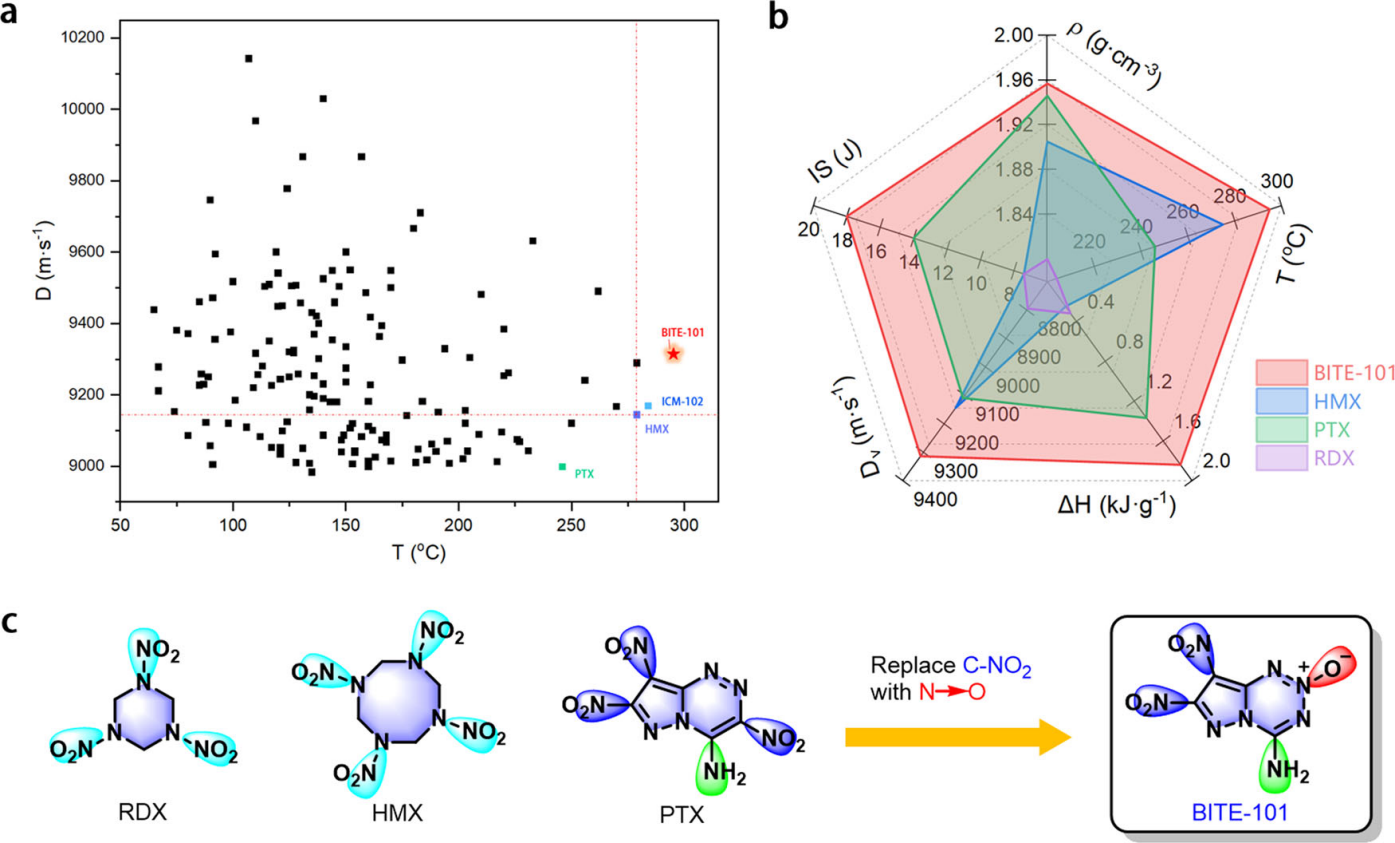
The properties comparison of BITE-101 with other HEDMs. **a** Detonation velocity vs decomposition temperature distributions chart of 152 selected neutral high explosives with detonation velocities >9000 m s^−1^; **b** The Radar chart of performance comparison for BITE-101 with HMX, PTX, and RDX; **c** The structures of HEDMs.

High energy density materials (HEDMs) present a class of materials that possess high densities and high detonation properties, since the existing contradiction between energy and safety, the pursuing of energetic compounds with good comprehensive properties is a desirable goal for researchers^1,2^. Energetic compounds, which are the major energy components in HEDMs, are structurally composed of backbones and explosophoric groups^3^. The explosophoric groups such as nitramine, azido, nitro, amino, N-O, nitroform etc. are the most commonly selected groups to increase the energy of energetic compounds. In terms of practical applications, a restricted process is required to examine their properties such as thermal stability, sensitivities, detonation velocity and pressure, etc., the worst performance of an energetic compound determines if it holds the application prospects^4,5^. The utilization of multi kinds of explosophoric groups to manipulate substitution positions of energetic compounds to regulate the properties of energetic compounds is a commonly used method. Octogen (HMX) is the most powerful military explosive in current use^6,7^, it has a density of 1.905 g cm^−3^, an onset decomposition temperature of 279 °C, detonation velocity of 9144 m s^−1^, and detonation pressure of 39.2 GPa. During the last few decades, lots of efforts have been devoted to synthesize CHON-based high explosives^8–16^. As shown in Fig. 1, 152 neutral energetic molecules with a detonation velocity >9000 m s^−1^ were collected and summarized. However, most of those high explosives decompose below 200 °C, which is difficult to meet the requirement for practical applications^4^. Furthermore, regards to the decomposition temperature, only 2,4,6-triamino-5-nitropyrimidine-1,3-dioxide (ICM-102)^17^ (*T*_d_ = 284 °C) was reported to have a higher decomposition compared to that of HMX to date. The search for new energetic compounds with superior performances that outperforms HMX remains a long-term challenge.

The construction of fused ring structures is an effective way to enhance the thermal stability of energetic compounds^16,18,19^. The incorporation of a nitro group or N-O group with an amino group to create novel energetic compounds with high density, excellent thermal stability as well as low sensitivity is a well-recognized strategy^19–26^. A representative fused energetic compound 3,7,8-trinitropyrazolo[5,1-c] [1,2,4] triazin-4-amine (PTX)^24^, which contains pyrazole-triazine fused ring and two kinds of explosophoric groups exhibit a high density of 1.946 g cm^−3^, the high decomposition temperature of 246 °C and comparable detonation properties to that of RDX^7^. Furthermore, the existing explosophoric groups of amino and nitro in PTX offers more possibility form hydrogen bonding interaction, therefore, giving better impact and friction sensitivity (*IS*: 14 J, *FS*: 324 N) than those of RDX (*IS*: 7.5 J, *FS*: 120 N) in which the nitramine is only explosophoric group in the structure. Maintaining the diversity of explosophoric groups in backbones is beneficial to achieve balanced comprehensive properties for energetic compounds.

Our continuing goal is to seek new energetic fused ring compounds with an ideal combination of high density, detonation properties, and acceptable sensitivity toward stimuli. We proposed an enhanced explosophoric group's cooperative strategy by replacing one C-NO_2_ of PTX with an N-O moiety, we envisioned that three different explosophoric groups could lead to diverse weak interactions while maintaining the zero-oxygen balance (CO as the product) and increasing the varieties of explosophoric group and also the heat of formation compared to that of PTX, hence make the newly designed compound with better detonation performances and higher stabilities than PTX. Herein, we present the synthesis of a novel fused ring energetic compound 4-amino-7,8-dinitropyrazolo-[5,1-*d*] [1,2,3,5]-tetrazine 2-oxide (named BITE−101), it was readily prepared by a three-step reaction. The structures and properties of prepared compounds were characterized by multinuclear NMR, infrared spectroscopy, elemental analysis, and differential scanning calorimetry (DSC). As expected, the more nitrogen atoms in the fused backbone and more kinds of explosophoric groups compared to those in PTX, affording BITE-101 a high density of 1.957 g cm^−3^, highest decomposition temperature (onset, 295 °C) among those CHON-based explosives with *D* > 9000 m s^−1^ (Fig. 1a). The comprehensive properties of BITE-101 are significantly better than those of the other three typical energetic materials (as shown in Fig. 1b). All of the above-mentioned features with the predicted detonation velocity of 9316 m s^−1^ and detonation pressure of 39.3 GPa demonstrating great potential for the application as a high energy density material.

## Results

### Materials synthesis

**Fig. 2:**
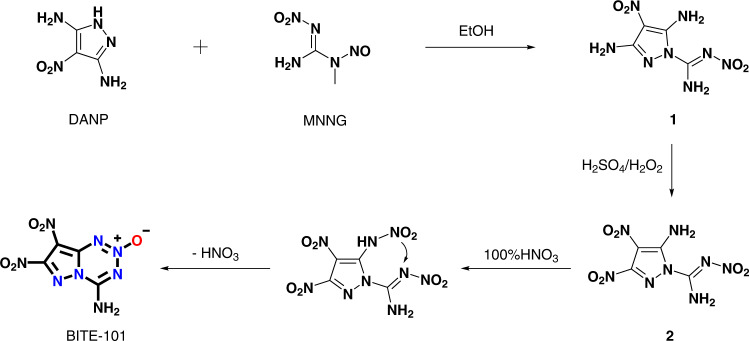
Synthetic route for BITE-101. BITE-101 was synthesized in three steps by starting from DANP and MNNG.

The synthesis of BITE-101 was commenced with the preparation of intermediate **1** by reacting 1-methyl-2-nitro-1-nitrosoguanidine^27^ and 4-nitro-1*H*-pyrazole-3,5-diamine^28^ in ethanol for 2 h under reflux conditions. The amino group on the 3-position of the pyrazole ring in **1** was selected and converted into a nitro group by using a mixture of 30% H_2_O_2_ and concentrated H_2_SO_4_, giving compound **2** with a yield of 45%. The treatment of **2** with 100% HNO_3_ resulting a cyclization reaction to the formation of fused 4-amino-7,8-dinitropyrazolo[5,1-d][1,2,3,5]-tetrazine 2-oxide (BITE-101) with a yield of 86% (Fig. 2). By comparing with other methods to construct [5,6]-fused 1,2,3,5-tetrazine-2-oxide backbones which always involves the generation of highly sensitive diazo intermediates^24,25,29^ or azides^22^ or cyanide and other highly toxic substances^20^, and also an additional *N*-oxidation step is required to access *N*-oxide^17,23^. Our new strategy to construct 1,2,3,5-tetrazine-2-oxide developed in this work is much safer and easier to be handled. The structure of BITE-101 and its intermediates were fully characterized by IR, ^1^H and ^13^C NMR spectroscopy as well as by elemental analysis and the spectra were listed in Supplementary Information.

### Single-crystal structure

**Fig. 3:**
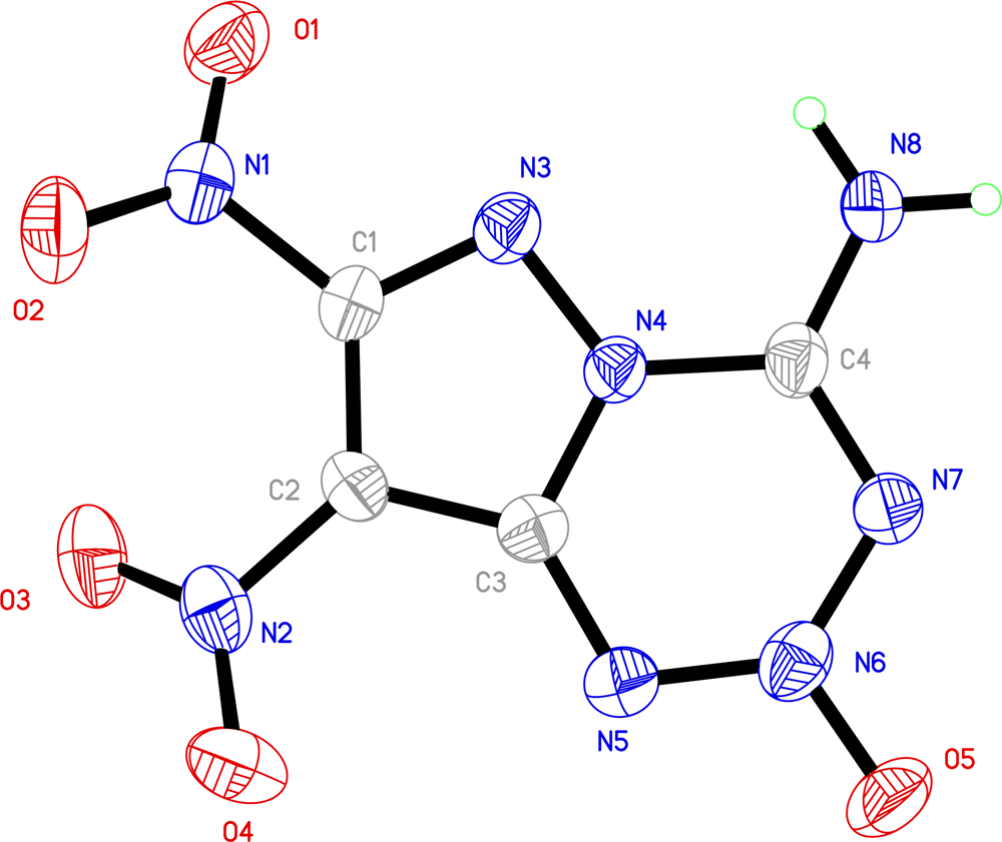
The crystal structure of BITE-101. The thermal ellipsoids are presented with 50% probability.

The structure of BITE-101 was further determined by an X-ray single-crystal diffraction study of the crystals grown from methanol solution at room temperature. As shown in Fig. 3, BITE-101 crystallizes in the orthorhombic P*2*_*1*_*2*_*1*_*2*_*1*_ space group, with four molecules per unit cell (*Z* = 4) and unit cell size V = 828.91(8) Å with a crystal density of 1.957 g cm^−3^ (298 K). The C-N bond length in the pyrazole fragment lies between 1.435–1.467 Å, which is similar to those in PTX (1.436–1.458 Å)^24^.

**Fig. 4:**
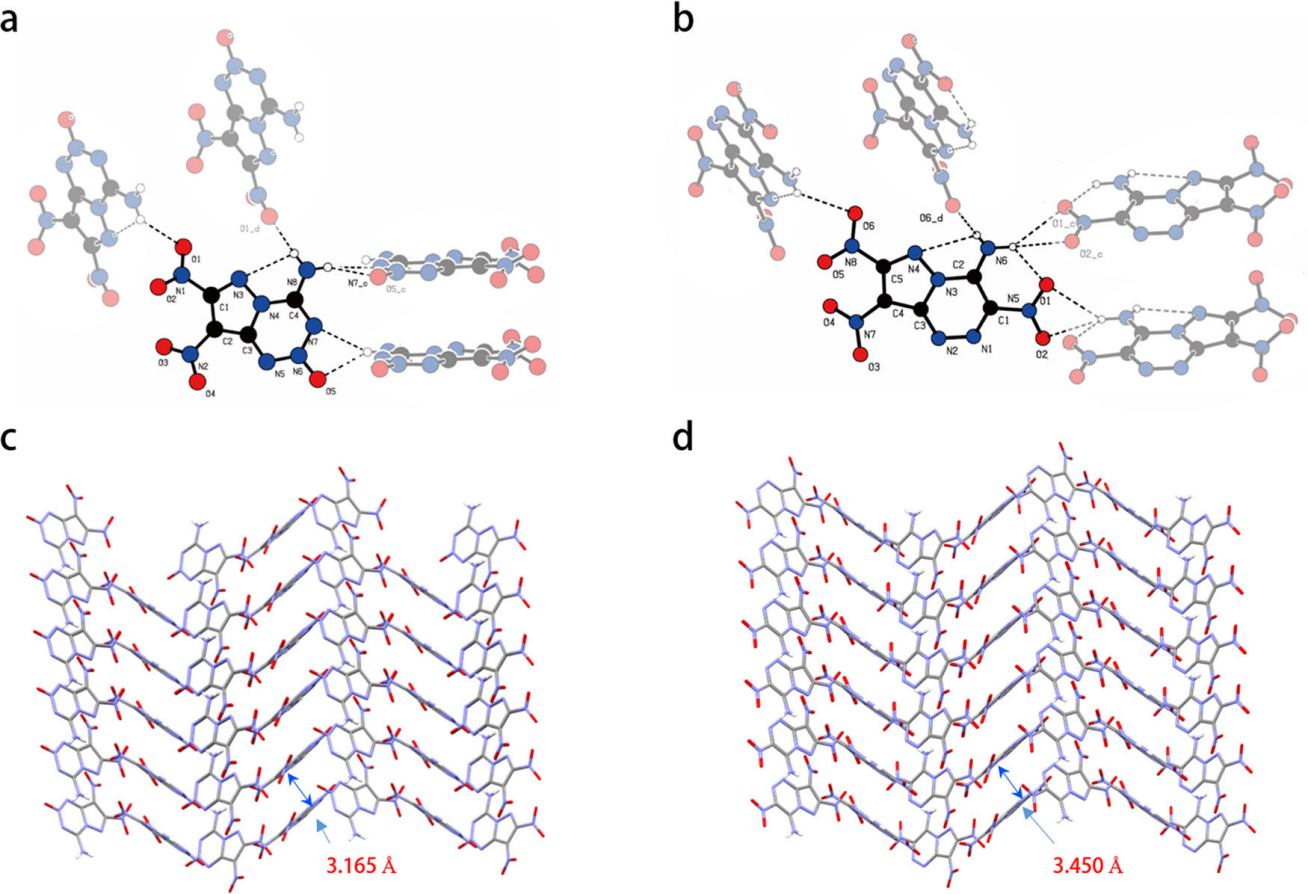
The intermolecular H-bonds of crystals of BITE−101 and PTX with their crystal packing. **a** The intermolecular H-bonds of BITE-101; **b** The intermolecular H-bonds of PTX; **c** The crystal stacking structure of BITE-101; **d** The crystal stacking structure of PTX.

While in the 1, 2, 3, 5-tetrazine-2-oxide motif, the C-N bond length in C-NH_2_ is 1.308 Å, the N-N bond length ranges from 1.328 to 1.356 Å, and the N-O bond length (1.240 Å) is shorter than N-O bond length in ICM-102^17^ (1.253(5) Å) and PTX (1.274(8) Å). In the packing diagram of BITE-101, four independent molecules in different orientations form a wave-like stacking structure, the interlayer spacing between adjacent parallel molecules of BITE-101 is 3.165 Å, which is shorter than that in PTX (3.450 Å) (Fig. 4c, d). The two nitro groups in BITE-101 forms a torsion angle of 14.71^o^, smaller than that in PTX (15.68^o^), the shorter average hydrogen bond length was also observed (BITE-101: 2.39 Å, PTX: 2.414 Å; Fig. 4a, b), all of above-resulting BITE-101 a higher density of 1.957 g cm^−3^.

### Thermostability

**Fig. 5:**
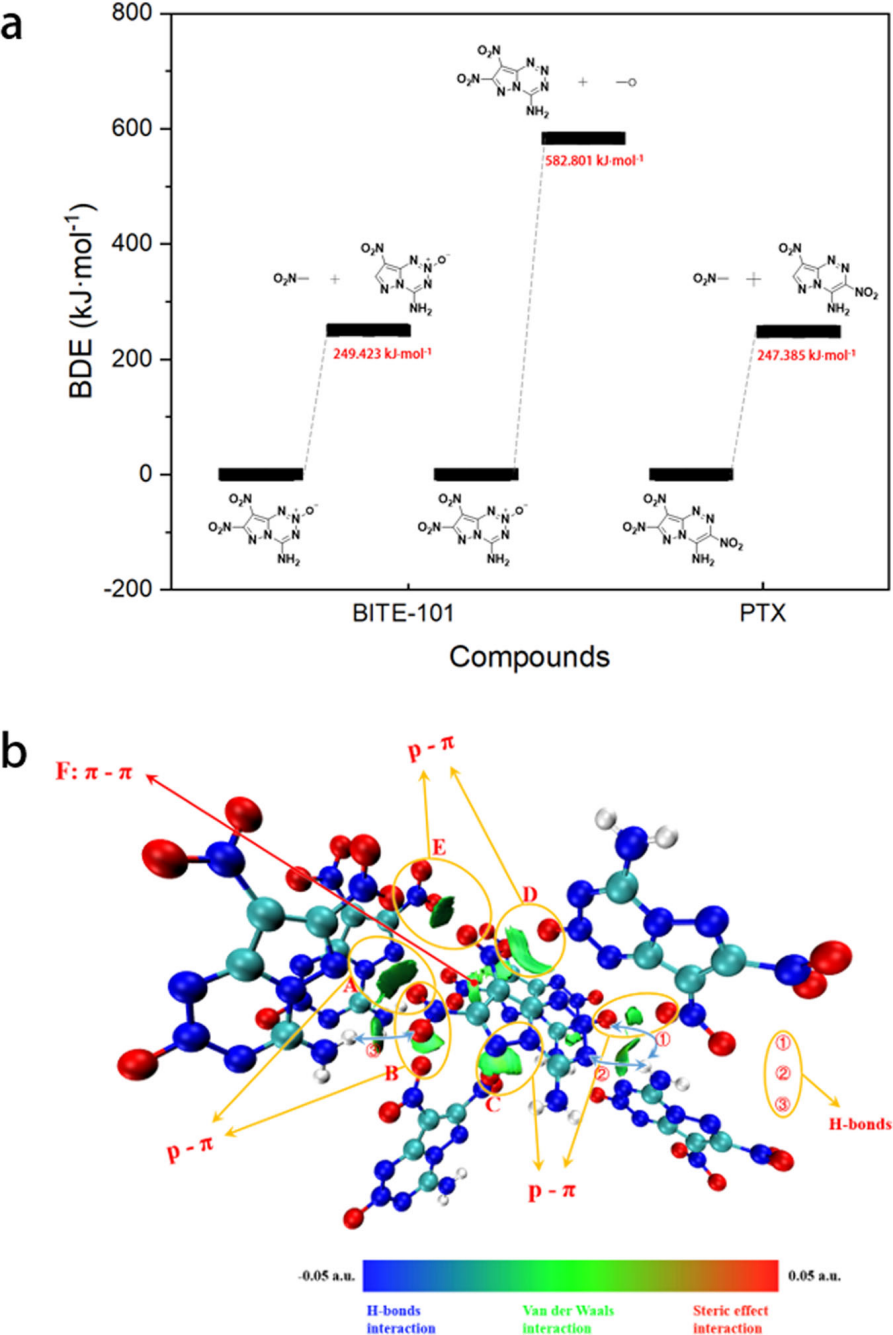
The BDE and weak interactions analyses for BITE-101. **a** The BDE of BITE-101 and PTX; **b** Intermolecular interactions analyses in BITE-101.

BITE-101 decomposes at 295 °C (onset) with a peak temperature at 300 °C (Supplementary Fig. 11), which is the highest among those reported CHON-based energetic compounds with detonation velocity >9000 m s^−1^ to date. To achieve a deeper understanding of the superior thermal stability of BITE-101, the Independent Gradient Model based on Hirshfeld partition (IGMH)^30^ and bond dissociation energy (BDE) calculations were performed. As shown in Fig. 5b, each molecule in BITE-101 form one intramolecular hydrogen bond and six inter-molecule hydrogen bonds with other molecules. Based on the IGMH analysis, the green isosurface between the central molecule and surrounding molecules fragment, the larger the area between atom pairs, the stronger the interaction, indicating strong π–π and p–π interactions existing in the structure. The bond dissociation energy (BDE) calculated using the Muitifwn software through the wave function information of the molecules^31^ for BITE-101 was calculated by comparing to those of PTX was shown in Fig. 5a (the detailed calculations were listed in Supplementary Table 6). The BDE value of the weakest bond in BITE-101 (C1-NO_2_: 249.423 kJ mol^−1^) is higher than that in PTX (C5-NO_2_: 247.385 kJ mol^−1^), demonstrating better thermal stability for BITE-101, which agrees well with the experimental results.

### Physicochemical properties

The heat of formation (HOF) of BITE-101 was calculated by using Gaussian 09 program^32^. The detailed calculations were provided in the Supplementary Information, and the result was listed in Table 1. Based on the measured density and calculated heat of formation for BITE-101, detonation velocity and pressure were predicted by using EXPLO5 v6.01^33^. Interestingly, BITE-101 gains a zero-oxygen balance which is the same as that of PTX and HMX when CO was used as the decomposition product. Though one less nitro group in the structure of BITE-101, possesses a much higher detonation velocity of 9316 m s^−1^ than that of PTX, the detonation performances of BITE-101 is also superior to those of HMX. Furthermore, the impact sensitivity of BITE-101 (*IS* = 18 J) is significantly better than that of HMX (*IS* = 7.4 J).

**Table 1:** Physicochemical Properties of BITE-101

Comp.	*T*_d_^a^(^o^C)	*ρ*^b^(g cm^−3^)	*D*_v_^c^(m s^−1^)	*P*^d^(GPa)	Δ_f_H^e^(kJ g^−1^)	OB^f^(%)	*N*(%)	*IS*^g^(J)	*FS*^h^(N)
BITE-101	295	1.957	9317	39.3	1.84	0	46.28	18	128
PTX^i^	246	1.946	9109	36.0	1.37	0	41.48	14	324
HMX^j^	279	1.905	9144	39.2	0.25	0	37.84	7.4	120

^a^Decomposition temperature (onset).

^b^Crystal density at 298 K.

^c^Detonation velocity calculated using EXPLO5 v6.01.

^d^Detonation pressure calculated using EXPLO5 v6.01.

^e^Calculated heat of formation.

^f^Oxygen balance based on CO for C_a_H_b_N_c_O_d_: OB (%) = 1600*(d-a-b/2)/Mw.

^g^Impact sensitivity evaluated by a standard BAM fallhammer.

^h^Friction sensitivity was evaluated by a BAM friction tester.

^i^Ref. 24.

^*j*^Refs. 7,17.

## Discussion

In summary, a high energy density compound BITE-101 was synthesized through a novel cyclization strategy in three steps without involving diazo, azide highly sensitive, or other toxic intermediates. The tri- explosophoric groups (C-NO_2_, C-NH_2_, N-O) in the structure of BITE-101 help to form extensive hydrogen bonding and π-π interactions and result in BITE-101 featuring a high measured density of 1.957 g cm^−3^, the highest decomposition temperature of 295 °C (onset) among CHON-based high explosives (*D* > 9000 m s^−1^) to date, better impact sensitivity (18 J) and friction sensitivity (128 N) along with a superior detonation velocity (9316 m s^−1^) to the benchmark explosive HMX, demonstrating great application prospects as a new generation of high energy density materials. The investigation of the application for BITE-101 is currently in progress in our lab.

## Methods

Caution! The prepared compounds are energetic materials and may explode under certain conditions. Appropriate safety precautions should be taken when preparing and handling.

### General

All reagents were purchased from Energy Chemical or Aladdin in analytical grade. DANP was prepared from the literature’s method^28^. ^1^H and ^13^C NMR spectra were recorded on Bruker AVANCE 400 nuclear magnetic resonance spectrometer. DMSO-d_6_ was employed as a solvent and locking solvent. Infrared (IR) spectra were recorded on an FT-IR spectrometer (Thermo Nicolet AVATAR 370). Decomposition (onset) temperature were recorded on a differential scanning calorimeter (DSC, TA Instruments discovery DSC25) at a scan rate of 5 °C min^−1^. Elemental analyses (C, H, N) were performed on a Vario Micro cube Elementar Analyzer. Impact and friction sensitivity measurements were made using a standard BAM Fallhammer and a BAM friction tester.

### Synthesis of 3, 5-diamino-*N'*,4-dinitro-1*H*-pyrazole-1-carboximidamide (1)

1-Methyl-2-nitro-1-nitrosoguanidine (MNNG, 2 g, 0.15 mol) was added to a solution of 3,5-diamino-4-nitro-pyrazole (DANP, 2 g, 0.20 mol) in 30 ml of ethanol, and the mixture was heated to 80 °C. After refluxing for 12 h, TLC (Solvent combination: EA:PE = 1:1; R_f_ = 0.55) detects that the reaction is completed. The precipitate was filtered off, washed with water, and dried in the air. The crude brownish-yellow solid of compound **1** was obtained (1.8 g, 55.9%). *T*_d_ (onset) 237 °C. ^1^H NMR (400 MHz, DMSO-d_6_) δ 9.09 (d, J = 159.5 Hz, 2H), 7.55(s, 2H), 5.90(s, 2H). ^13^C NMR (101 MHz, DMSO-d_6_) δ 155.4, 150.9, 148.5, 109.1 ppm. IR (KBr) ν̃ = 3477, 3553, 3234, 1646, 1611, 1568, 1519, 1490, 1353, 1253, 1203, 1168, 1137, 1047, 954, 820, 798, 770, 690, 647, 612, 566, 543, 409 cm^−1^. Elemental analysis (%) for C_4_H_6_N_8_O_4_ (230.05) Calcd: C 20.88, H 2.63, N 48.69. Found: C 20.83, H 2.39, N 49.02.

### Synthesis of 5-amino-3, 4-trinitro-1*H*-pyrazole-1-carboximidamide (2)

About 10 mL of 30% H_2_O_2_ was added into a 100 ml flask and cooled by an ice-salt bath, 10 mL of 60% H_2_SO_4_ was added dropwise while maintaining the temperature <0 °C. Then 3, 5-diamino-*N'*, 4-dinitro-1*H*-pyrazole-1-carboximidamide (**1**, 1.15 g, 5 mmol) was added to the mixed solution at 0 °C in batches after the addition was completed. The reaction temperature gradually rose to room temperature and reacted for another 3 h. After the reaction was completed, which was monitored by TLC (Solvent combination: EA:PE = 1:2; R_f_ = 0.40), the reaction solution was poured into ice water, and a yellow solid is precipitated. The precipitate was collected by filtration and washed with water to give **2** (0.58 g, 34.6%). *T*_d_ (onset) 204 °C. ^1^H NMR (400 MHz, DMSO-d_6_) δ 9.88 (s, 1H), 8.82 (s, 1H). ^13^C NMR (101 MHz, DMSO-d_6_) δ 155.1, 150.2, 148.5, 109.0 ppm. IR (KBr) ν̃ =3439, 3374, 3304, 3248, 1655, 1565, 1530, 1504, 1451, 1434, 1377, 1346, 1313, 1255, 1214, 1172, 1085, 1034, 948, 879, 820, 783, 773, 762, 756, 715, 683, 674, 624, 517, 462 cm^−1^. Elemental analysis (%) for C_4_H_4_N_8_O_6_ (260.03) Calcd: C 18.47, H 1.55, N 43.08. Found: C 18.16, H 1.89, N 42.71.

### Synthesis of 4-amino-7,8-dinitropyrazolo[5,1-*d*][1,2,3,5]-tetrazine 2-oxide (BITE-101)

To a 50 mL flask containing 3 ml 100% HNO_3_ cooled to 0 °C was added 100 mg Compound **2** (100 mg, 0.384 mmol) in batches, after then, the reaction mixture was slowly warmed up to room temperature and stirred for another 3 h. After the reaction was completed it was monitored by TLC (Solvent combination: EA:PE = 1:1; R_f_ = 0.5). Then, the reaction mixture was poured into ice water to precipitate a yellow solid. The solid was collected by filtration and washed with water to obtain a light yellow solid powder of BITE-101 (79.9 mg, 85.9%). *T*_d_ (onset) 295 °C. ^1^H NMR (400 MHz, DMSO-d_6_) δ 10.21 (d, J = 9.1 Hz, 2H). ^13^C NMR (101 MHz, DMSO) δ 152.8, 151.7, 146.2, 110.2 ppm. IR (KBr) ν̃ = 3344, 3247, 3194, 1660, 1594, 1561, 1507, 1472, 1417, 1390, 1376, 1359, 1333, 1262, 1233, 1136, 989, 879, 855, 812, 778, 767, 735, 719, 713, 660, 643, 608, 580, 549, 460 cm^−1^. Elemental analysis (%) for C_4_H_2_N_8_O_5_ (242.01) Calcd: C 19.84, H 0.83, N 46.28. Found: C 19.43, H 1.27, N 46.64.

## Supplementary information

Supplementary Information

Peer Review File

## Data Availability

All relevant data were included within this article and Supplementary Information. The X-ray crystallographic coordinates for BITE−101 reported in this study have been deposited at the Cambridge Crystallographic Data Centre (CCDC), under deposition number 2175186. These data can be obtained free of charge from The Cambridge Crystallographic Data Centre via www.ccdc.cam.ac.uk/data_request/cif. The data that support this study are available from the corresponding author upon reasonable request.

## References

[1] Klapötke, T. M. *High Energy Density Materials* (Springer, 2007).

[2] Badgujar DM, Talawar MB, Asthana SN, Mahulikar PP (2008). Advances in science and technology of modern energetic materials: an overview. J. Hazard. Mater..

[3] Pagoria PF, Lee GS, Mitchell AR, Schmidt RD (2002). A review of energetic materials synthesis. Thermochim. Acta.

[4] Brinck, T. *Green Energetic Materials* (John Wiley & Sons Inc, 2014).

[5] Klapötke, T. M. *Chemistry of High-Energy Materials* (De Gruyter, 2019).

[6] Agrawal, J. P. & Hodgson, R. *Organic Chemistry of Explosives* (John Wiley & Sons, 2007).

[7] Meyer, R., Köhler, J. & Homburg, A. *Explosives* (Wiley, 2016).

[8] Yin P, Zhang Q, Shreeve JM (2016). Dancing with energetic nitrogen atoms: versatile N-functionalization strategies for *N*-heterocyclic frameworks in high energy density materials. Acc. Chem. Res..

[9] He C, Tang Y, Mitchell LA, Parrish DA, Shreeve JM (2016). *N*-Oxides light up energetic performances: synthesis and characterization of dinitraminobisfuroxans and their salts. J. Mater. Chem. A.

[10] Zhang J, Yin P, Mitchell LA, Parrish DA, Shreeve JM (2016). N-functionalized nitroxy/azido fused-ring azoles as high-performance energetic materials. J. Mater. Chem. A.

[11] Zhang W (2017). A promising high-energy-density material. Nat. Commun..

[12] Yin P, Parrish DA, Shreeve JM (2015). Energetic multifunctionalized nitraminopyrazoles and their ionic derivatives: ternary hydrogen-bond induced high energy density materials. J. Am. Chem. Soc..

[13] Zhang G, Yi Z, Cheng G, Yang W, Yang H (2022). Polynitro-functionalized azopyrazole with high performance and low sensitivity as novel energetic materials. ACS Appl. Mater. Interfaces.

[14] Fei T (2022). New role for an *N*,*N*′-alkylidene bridge: taming the hygroscopicity of acidic energetic materials with enhanced stability. Cryst. Growth Des..

[15] Cai J (2022). High performance and heat-resistant pyrazole-1,2,4-triazole energetic materials: tuning the thermal stability by asymmetric framework and azo-bistriazole bridge. Chem. Eng. J..

[16] Gao H, Zhang Q, Shreeve JM (2020). Fused heterocycle-based energetic materials (2012–2019). J. Mater. Chem. A.

[17] Wang Y (2018). Accelerating the discovery of insensitive high-energy-density materials by a materials genome approach. Nat. Commun..

[18] Ma Q (2022). Accelerated discovery of thermostable high-energy materials with intramolecular donor–acceptor building blocks. Chem. Commun..

[19] Feng Y (2020). Construction of an unusual two-dimensional layered structure for fused-ring energetic materials with high energy and good stability. Engineering.

[20] Chen S, Zhang W, Wang Y, Zhang Q (2021). [1,2,4]Triazolo[4,3-b]pyridazine as a building block towards low-sensitivity high-energy materials. Chem. Eng. J..

[21] Lei C, Cheng G, Yi Z, Zhang Q, Yang H (2021). A facile strategy for synthesizing promising pyrazole-fused energetic compounds. Chem. Eng. J..

[22] Huang W, Tang Y, Imler GH, Parrish DA, Shreeve JM (2020). Nitrogen-rich tetrazolo[1,5-*b*]pyridazine: promising building block for advanced energetic materials. J. Am. Chem. Soc..

[23] Wei H, Zhang J, Shreeve JM (2015). Synthesis, characterization, and energetic properties of 6-amino-tetrazolo[1,5-b]-1,2,4,5-tetrazine-7-*N*-oxide: a nitrogen-rich material with high density. Chem. Asian J..

[24] Schulze MC, Scott BL, Chavez DE (2015). A high density pyrazolo-triazine explosive (PTX). J. Mater. Chem. A.

[25] Piercey DG, Chavez DE, Scott BL, Imler GH, Parrish DA (2016). An energetic triazolo-1,2,4-triazine and its N-oxide. Angew. Chem. Int. Ed..

[26] Deng M (2022). From the sensitive primary explosive ICM-103 to insensitive heat-resistant energetic materials through a local azide-to-amino structural modification strategy. Chem. Eng. J..

[27] Xu Z (2017). A facile and versatile synthesis of energetic furazan‐functionalized 5‐nitroimino‐1,2,4‐triazoles. Angew. Chem. Int. Ed..

[28] Guillard J, Goujon F, Badol P, Poullain D (2003). New synthetic route to diaminonitropyrazoles as precursors of energetic materials. Tetrahedron Lett..

[29] Creegan SE, Piercey DG (2020). Nitroacetonitrile as a versatile precursor in energetic materials synthesis. RSC Adv..

[30] Lu T, Chen Q (2022). Independent gradient model based on Hirshfeld partition: a new method for visual study of interactions in chemical systems. J. Comput Chem..

[31] Lu T, Chen F (2012). Multiwfn: a multifunctional wavefunction analyzer. J. Comput. Chem..

[32] Lipparini, F. et al. Gaussian 09, revision E. 01. (Gaussian Inc., 2013).

[33] Sućeska, M. *EXPLO5 6.01* (Brodarski Institute, 2013).

