# Supplementary material for: Tri-explosophoric groups driven fused energetic heterocycles featuring superior energetic and safety performances outperforms HMX
Source: Nat Commun. 2022 Sep 28;13:5697. doi: 10.1038/s41467-022-33413-7 (PMC9519884; doi:10.1038/s41467-022-33413-7)
Supplement: Supplementary file 1 — Supplementary Information [file 41467_2022_33413_MOESM1_ESM.pdf]

Supplementary Information

Tri-explosophoric groups driven fused energetic compounds featuring superior performances outperforms HMX

Jie Li[1], Yubing Liu[1,2,3], Wenqi Ma[1], Teng Fei[1,2], Chunlin He[1,2,3,4]*, Siping Pang[1,2]*

[1]Experimental Center of Advanced Materials, School of Materials Science & Engineering, Beijing Institute of Technology, Beijing, 10081 (China). [2]State Key Laboratory of Explosion Science and Technology, Beijing Institute of Technology, Beijing, 10081 (China). [3]Yangtze Delta Region Academy of Beijing Institute of Technology, Jiaxing 314019, China. [4]Chongqing Innovation Center, Beijing Institute of Technology, Chongqing 401120, China. Corresponding authors: Email: chunlinhe@bit.edu.cn (C. He); pangsp@bit.edu.cn (S. Pang).

Table of Contents

1 Theoretical calculations 1

2 X-ray data, calculations and spectra for NMR, DSC and IR 2

2.1 X-Ray crystallography 2

2.2 Calculation details of ESP and BDE 4

2.3 NMR spectra 5

2.4 DSC plots 8

2.5 IR plots 8

3 The 152 selected neutral high-energy explosives with detonation velocities above 9000 m·s^{-1} 9

4 Supplementary References 24

1 Theoretical calculations

The calculations of the gas phase enthalpies of formation were carried out using Gaussian 09 (Revision D.01) suite of programs[1] based on isodemic reactions (**Supplementary Scheme 1**). The geometric optimization and frequency analyses of the structures were calculated using B3LYP/6-31+G** level. The gas phase enthalpy of formation was computed and the enthalpy of reaction was obtained by combining the M062X/def2TZVPP[2] energy difference for the reactions, the scaled zero point energies (*ZPE*), values of thermal correction (*Hr*), and other thermal factors. The solid state heat of formation were calculated

using G2 level and calculated with Trouton's rule according to equation (1)[3] (T represents either the melting point or the decomposition temperature when no melting occurs prior to decomposition)

$$\Delta H_{sub} = 188/J \cdot mol^{-1} \cdot K^{-1} \times T \qquad (1)$$

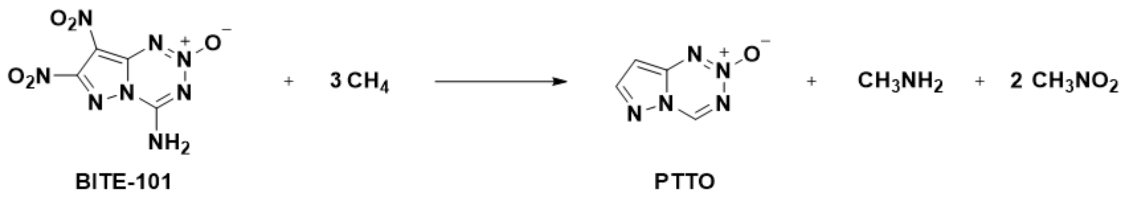

Supplementary Fig. 1. Isodesmic reaction for BITE-101

Supplementary Table 1. the scaled zero point energies (*ZPE*), values of thermal correction (H_r), total energy (E_0) and heats of formation (*HOF*)

Species	*ZPE*	H_r	E_0	Corrected E_0	*HOF* (kJ·mol^{-1})
BITE-101	0.106736	0.121274	-967.438377	-967.32000	551.2085976
PTTO	0.085555	0.093031	-503.0629179	-502.97221	559.4042878
CH_4	0.044604	0.048417	-40.501863	-40.45465	-74.6
CH_3NH_2	0.063823	0.068176	-95.845222	-95.77878	-23.5
CH_3NO_2	0.049663	0.05408	-245.010369	-244.95763	-81

2 X-ray data, calculations and spectra for NMR, DSC and IR

2.1 X-Ray crystallography

Supplementary Table 2. Crystal data and structure refinement for BITE-101.

	BITE-101
Formula	$C_4H_2N_8O_5$
Mw [g mol^{-1}]	242.14
T [K]	298.0
Crystal system	Orthorhombic
Space group	P212121
a[Å]	4.9834(3)
b[Å]	8.1806(4)
c[Å]	20.1627(13)
α[o]	90
β[o]	90

γ[o]	90
V [Å 3]	821.98(8)
Z	4
ρcalc[g cm^{-3}]	1.957
μ [mm^{-1}]	0.178
F(000)	488.0
2θ range [o]	4.04-52.738
Reflections collected	5683
Rint	0.0532
Data/restraints/parameters	1627 / 0 / 154
Final R index [I>2σ(I)]	R 1 =0.0436
	wR 2 =0.0799
Final R index [all data]	R 1 =0.0704
	wR 2 =0.0943
GOF on F2	1.109
CCDC number	2175186

Supplementary Table 3. Selected bond lengths [Å] for compound BITE-101

bond	length/Å	bond	length/Å
O1-N1	1.219(5)	N5-N6	1.328(4)
O5-N6	1.240(4)	N5-C3	1.343(5)
O3-N2	1.222(5)	O4-N2	1.222(5)
N4-N3	1.361(4)	N2-C2	1.435(5)
N4-C3	1.367(5)	N6-N7	1.356(5)
N4-C4	1.375(5)	N7-C4	1.326(5)
O2-N1	1.210(4)	N8-C4	1.308(5)
N3-C1	1.319(5)	C3-C2	1.390(5)
N1-C1	1.467(5)	C2-C1	1.394(6)

Supplementary Table 4. Selected bond angles [°] for compound BITE-101

bond	angle/(˚)	bond	angle/(˚)
N3-N4-C3	115.2(3)	C4-N7-N6	117(3)
N3-N4-C4	124.5(3)	N4-C3-C2	103.9(3)
C3-N4-C4	120.2(3)	N5-C3-N4	122.5(3)
C1-N3-N4	101.5(3)	N5-C3-C2	133.5(4)
O1-N1-C1	116.9(3)	N7-C4-N4	118.8(4)
O2-N1-O1	125.4(4)	N8-C4-N4	119.3(4)
O2-N1-C1	117.5(4)	N8-C4-N7	121.9(3)
N6-N5-C3	113.3(3)	C3-C2-N2	124.9(4)
O3-N2-O4	125.3(4)	C3-C2-C1	104.8(3)
O3-N2-C2	117.3(4)	C1-C2-N2	129.9(4)

O4-N2-C2	117.4(4)	N3-C1-N1	115.6(4)
O5-N6-N5	117.6(3)	N3-C1-C2	114.6(3)
O5-N6-N7	114.2(3)	C2-C1-N1	129.4(3)
N5-N6-N7	128.2(3)		

Supplementary Table 5. Hydrogen bonds present in compound BITE-101

D-H…A	d(D-H)/Å	d(H…A)/Å	d(D…A)/Å	<(DHA)/o	comment
N8-H8A…O5	0.86	2.33	3.101(4)	149	inter
N8-H8A…N7	0.86	2.44	3.216(5)	151	inter
N8-H8B…N3	0.86	2.47	2.787(5)	103	intra
N8-H8B…O1	0.86	2.32	3.033(5)	141	inter

2.2 Calculation details of ESP and BDE

Electrostatic Potential (ESP)

To obtain the ESP of compounds, gas state molecule geometries were optimized at the theory level of M062X/6-311G(d,p) through Gaussian 09 (Revision D.01) suite of program[4]. Then, ESPs at 0.001 a. u. and minimum values on the surfaces were calculated and visualized by Multiwfn and VMD programs using check file as input[5,6].

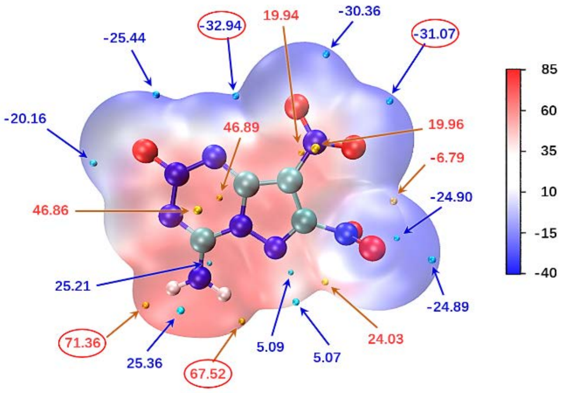

Supplementary Fig. 2 Electrostatic potential of BITE-101.

Bond Dissociation Energy

Supplementary Table 6. Bond Dissociation Energy of BITE-101, PTX and TNDPT.

Comp.	Bond type	BDE (UB3LYP/6-31G*/kJ·mol)	T_d (°C)	IS(J)
BIT-101	C1-NO_2	249.423	295	18
	C2-NO_2	273.052		
PTX	C1-NO_2	264.183	246	13.23
	C4-NO_2	264.842		

	C5-NO_2	247.385		
TNDPT	C1-NO_2	245.069	233	10
	C2-NO_2	241.262		
	C5-NO_2	241.259		

2.3 NMR spectra

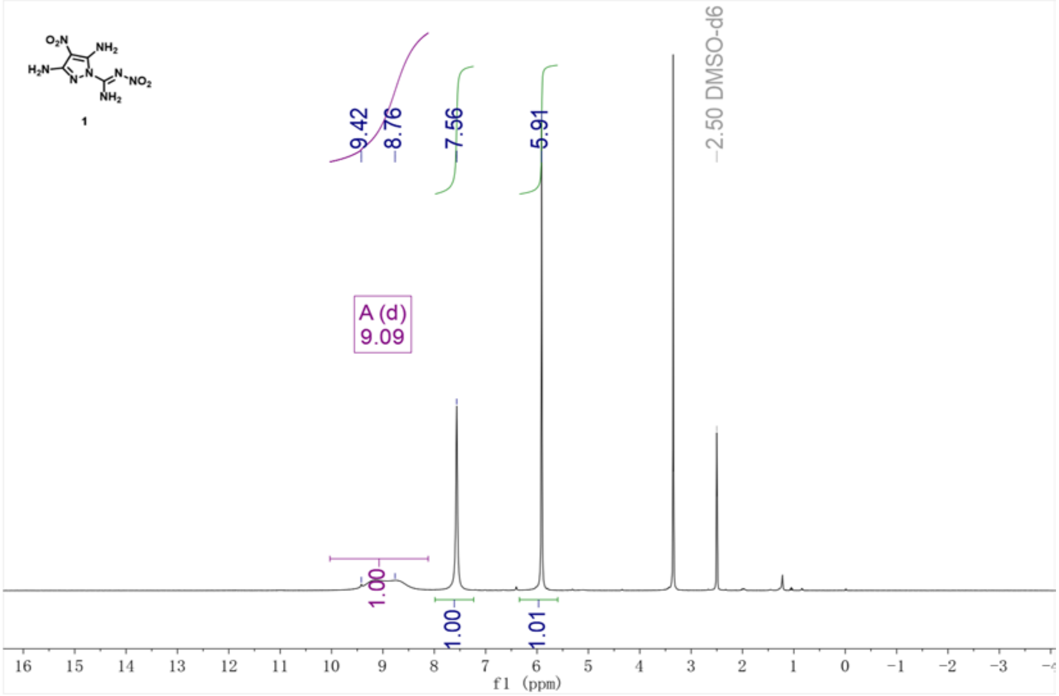

Supplementary Fig. 3 ^{1}H NMR spectrum of **1**

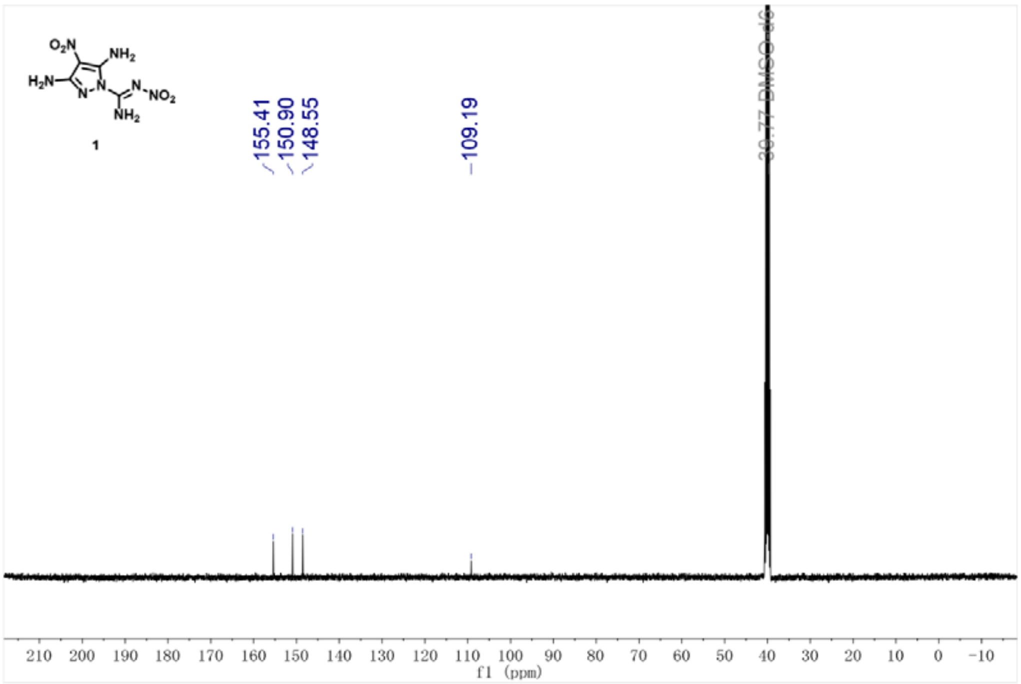

Supplementary Fig. 4 ^{13}C NMR spectrum of **1**

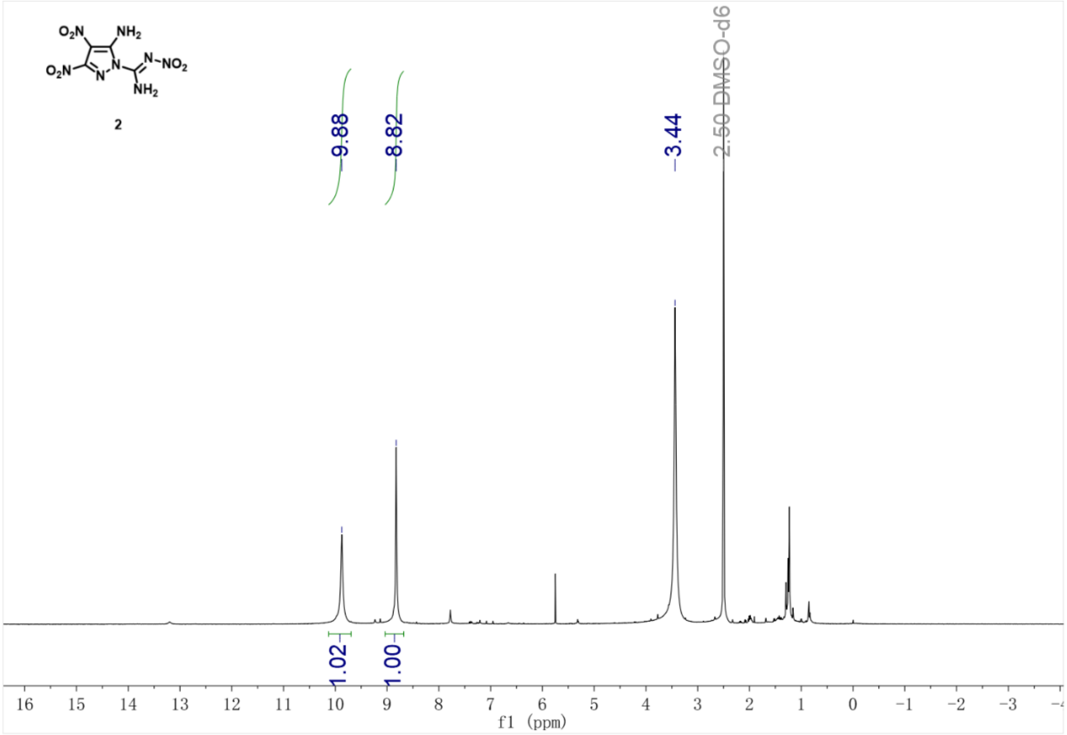

Supplementary Fig.5 ^{1}H NMR spectrum of **2**

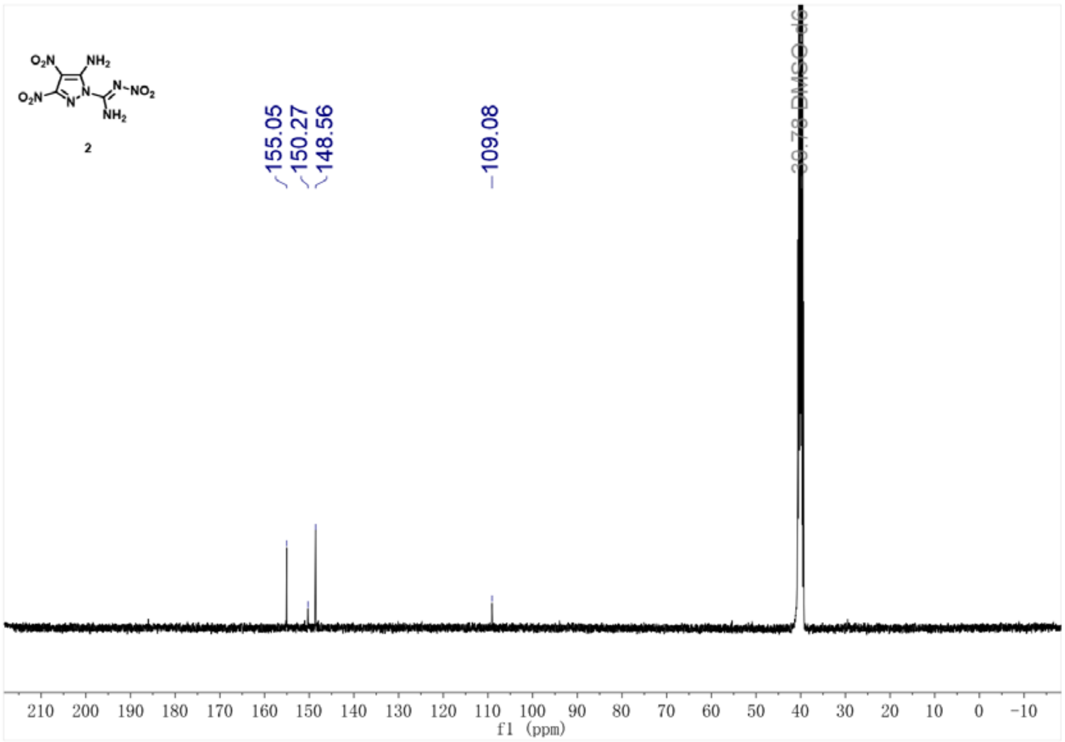

Supplementary Fig.6 ^{13}C NMR spectrum of **2**

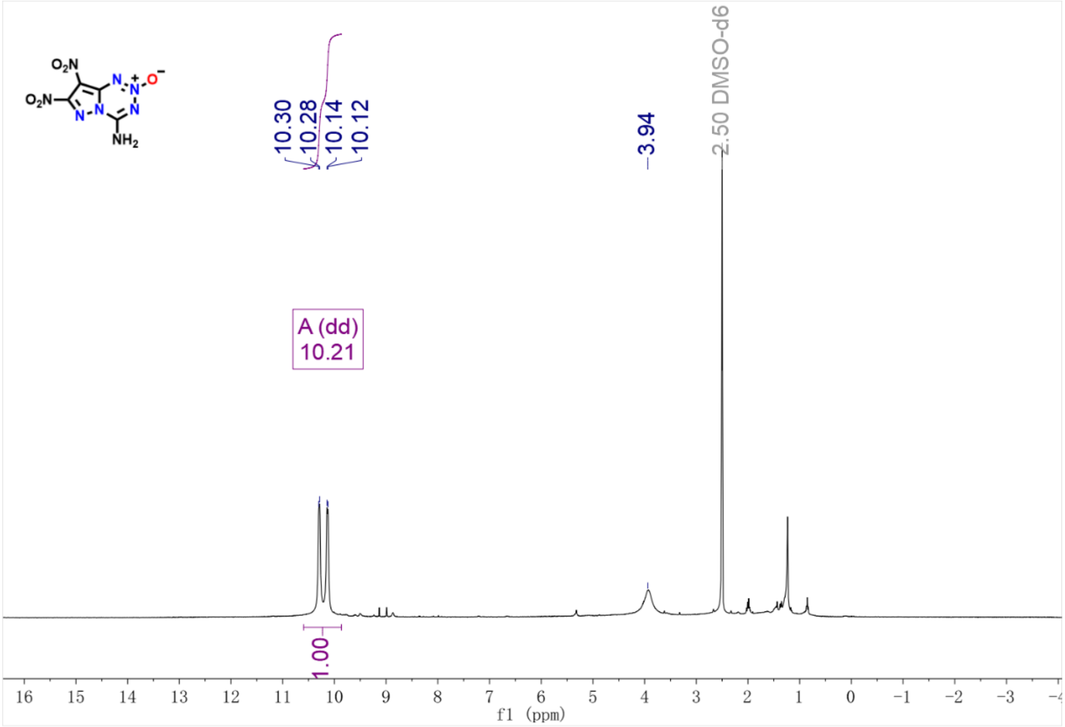

Supplementary Fig.7 ^{1}H NMR spectrum of BITE-101

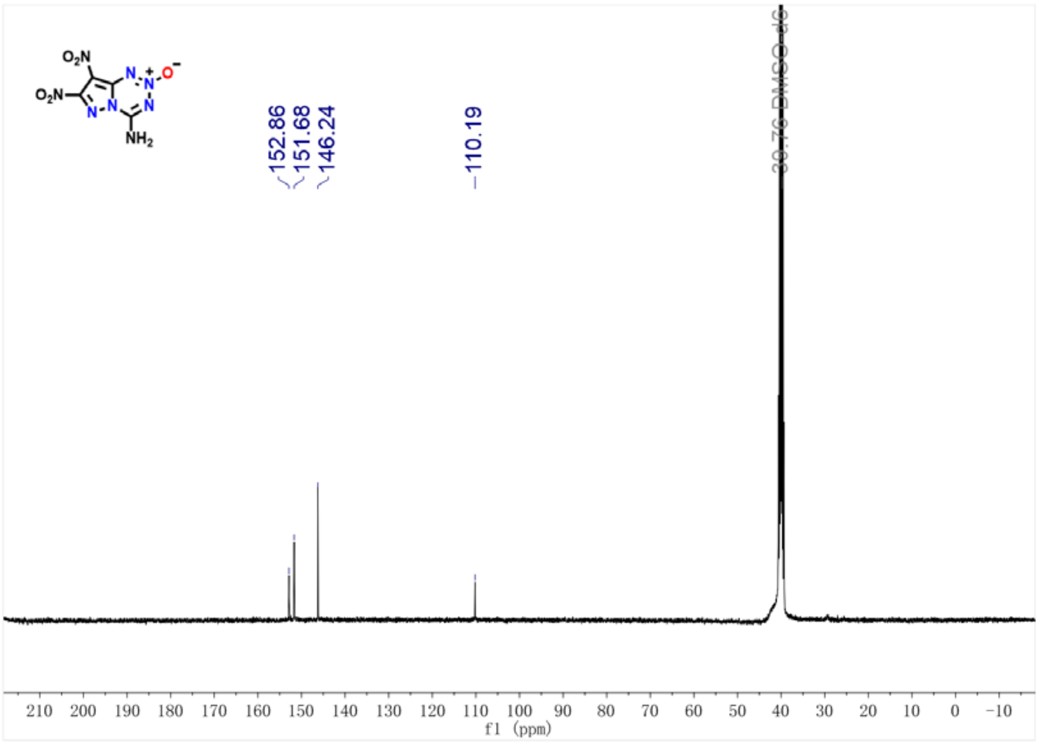

Supplementary Fig. 8 ^{13}C NMR spectrum of BITE-101

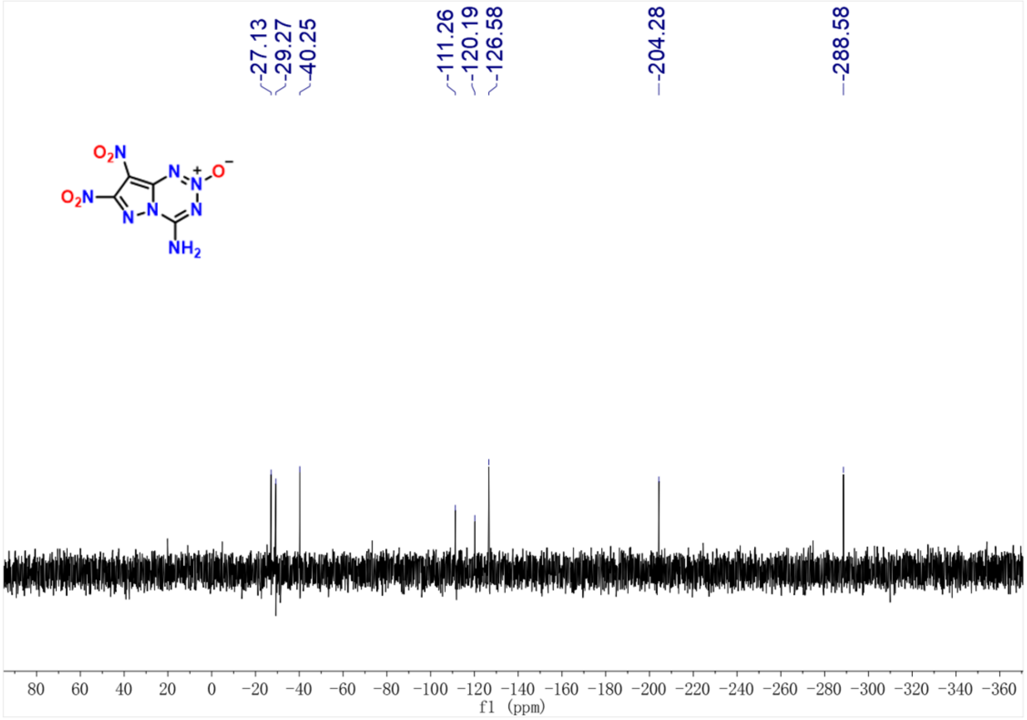

Supplementary Fig. 9. ^{15}N NMR spectrum of BITE-101

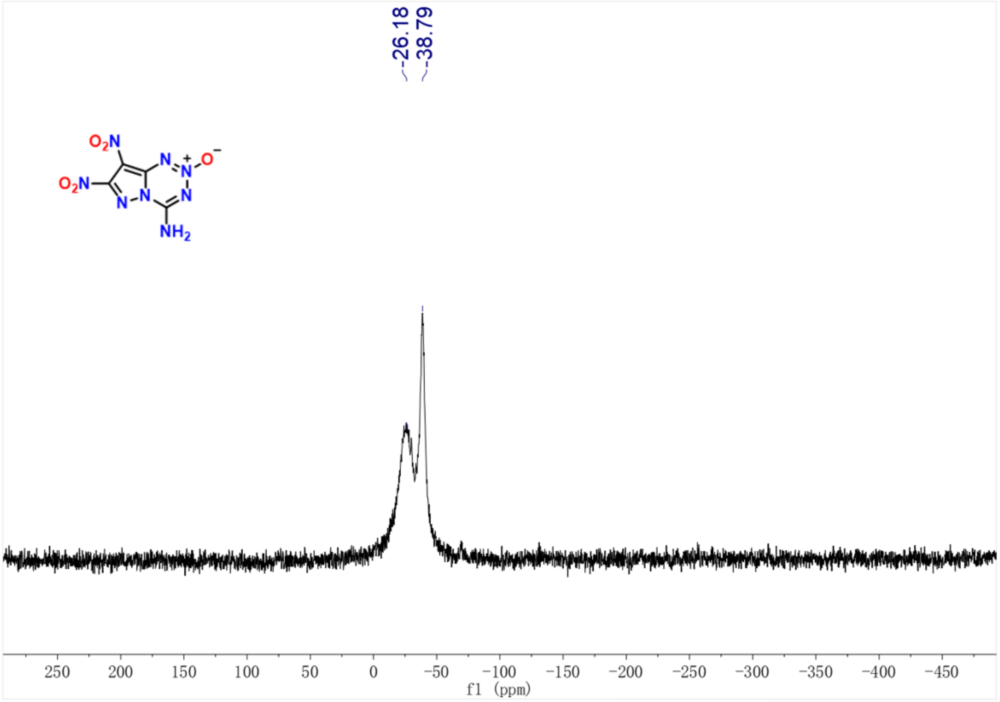

Supplementary Fig. 10 ^{14}N NMR spectrum of BITE-101

2.4 DSC plots

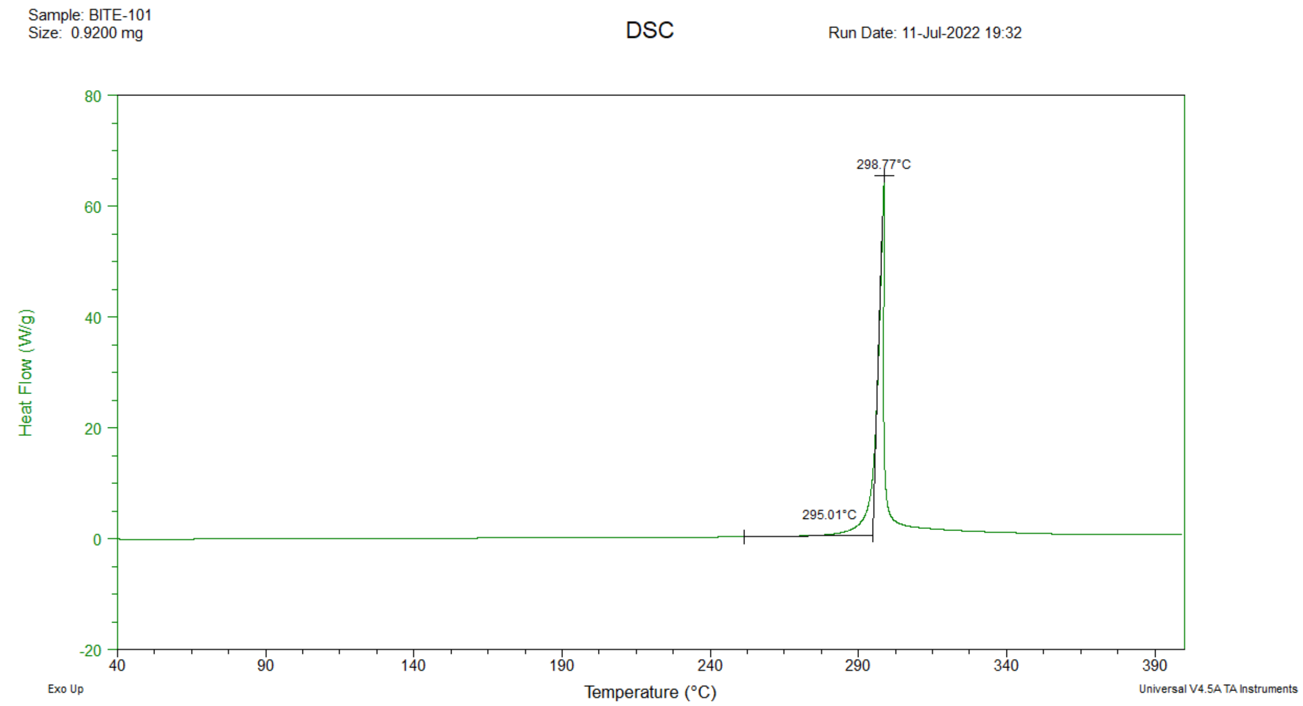

Supplementary Fig. 11 DSC curve of BITE-101

2.5 IR plots

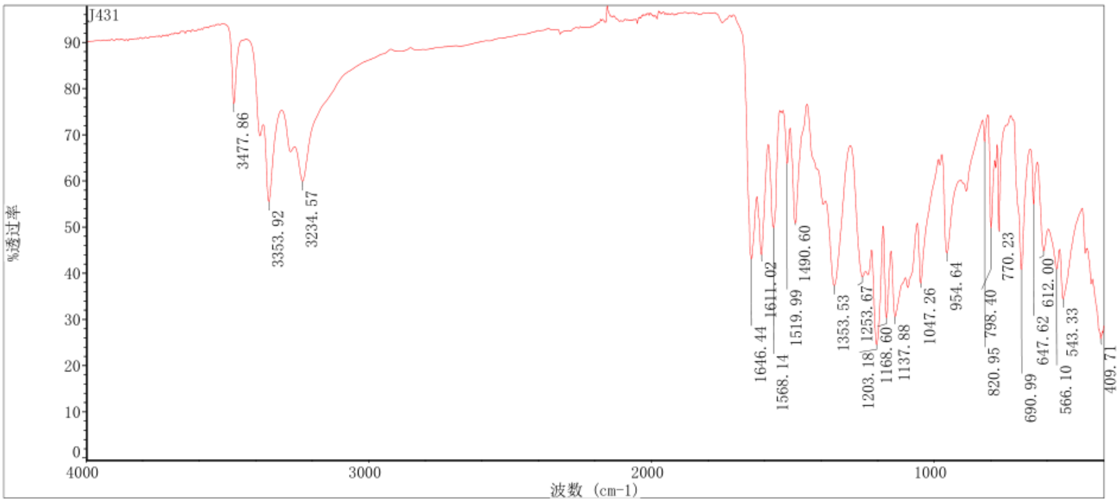

Supplementary Fig.12 IR curve of **1**

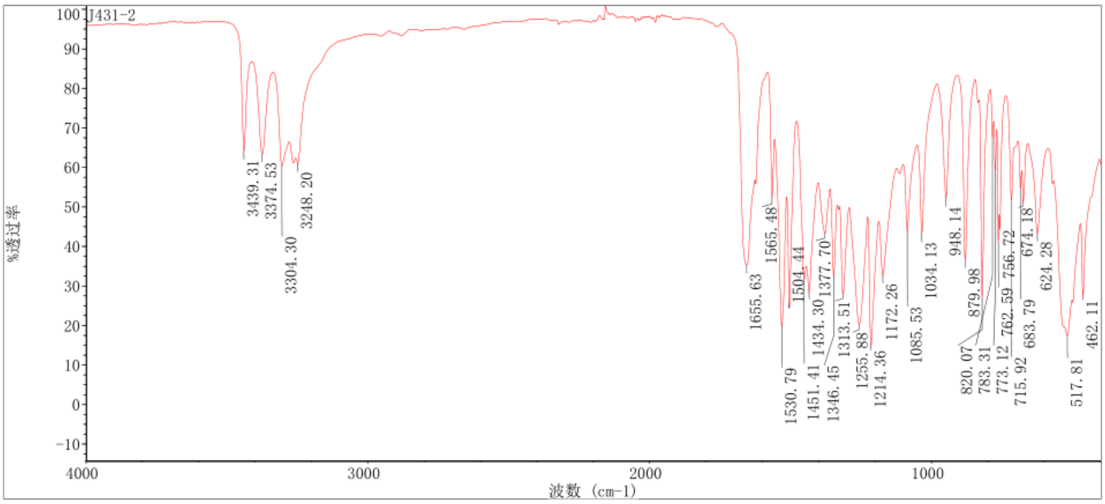

Supplementary Fig.13 IR of **2**

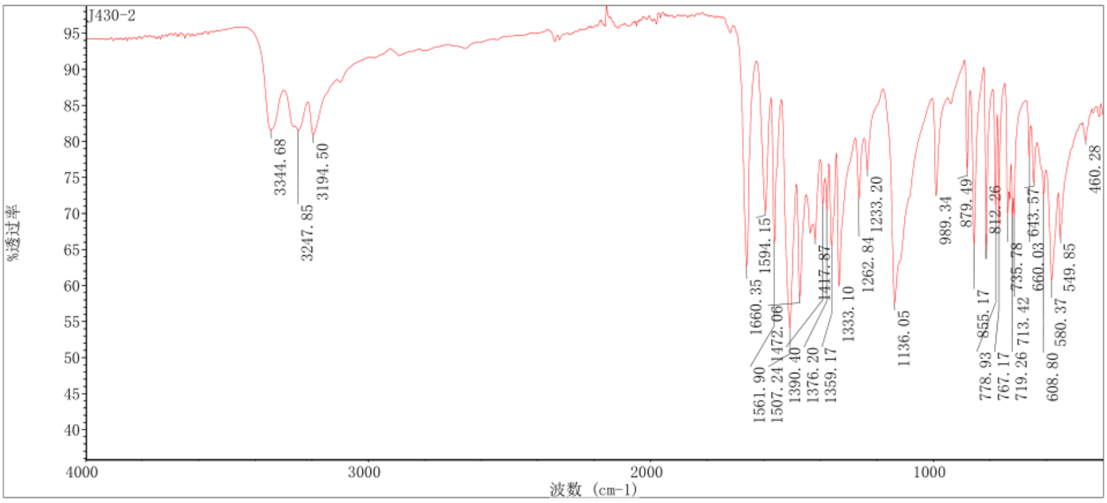

Supplementary Fig.14 IR of BITE-101

3 The 152 selected neutral high-energy explosives with detonation velocities above 9000 m·s^{-1}

Supplementary Table 7. The 152 selected neutral high-energy explosives with detonation velocities above 9000 m·s^{-1} .

Structure	d (g·cm^{-3})	T_d (°C)	D (m·s^{-1})	P (GPa)	IS (J)	FS (N)	Ref.
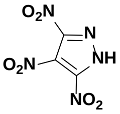	1.87	220	9253	38.6	17	92	7

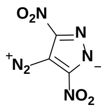	1.81	154	9038	35	2.5	<5	[8]
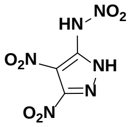	1.97	135	9430	41.6	4	40	[9,10]
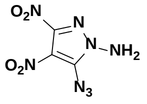	1.82	121	9053	35.8	1.5	5	[11]
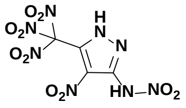	1.9	124	9124	37.2	5	80	[12]
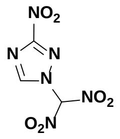	1.85	131	9050	37	7	252	[13]
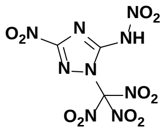	1.97	121	9033	34.2	4	144	[14]
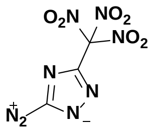	1.89	127	9317	37.6	<1	5	[15]
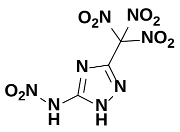	1.91	125	9250	36.9	2	40	[16]
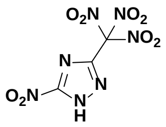	1.94	135	8983	35.5	9	--	[17,18]
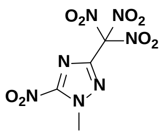	1.88	153	9006	36.9	13	--	[18]

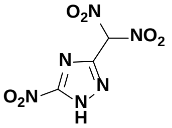	1.91	87	9229	38.4	9.5	--	[18]
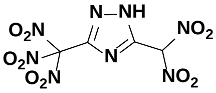	1.97	143	9180	38.7	6	120	[19]
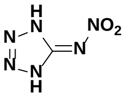	1.87	122	9450	39.4	1.5	8	[20,21]
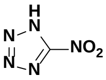	1.9	130	9457	39	<1	<5	[22,23]
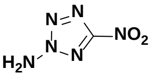	1.79	140	9087	36.8	<1	<5	[24]
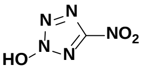	1.94	120	9447	40.4	N.D	N.D.	[25]
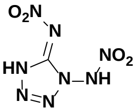	1.93	110	9967	43.4	1	<5	[26]
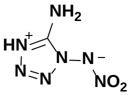	1.84	85	9460	40.3	<0.25	<5	[27]
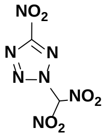	1.97	109	9220	38.5	5	80	[13]
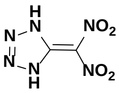	1.87	92.2	9594	40.3	3	28	[28,29]
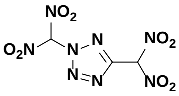	1.92	88	9123	37.7	3	120	[30]

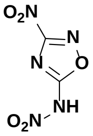	1.88	219	9095	37.7	15	--	[31]
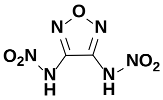	1.9	99	9376	40.5	<1	<5	[32]
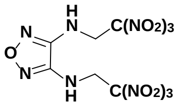	1.83	160	8998	36.3	7.1	108	[33]
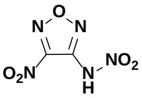	1.94	65	9438	39.8	4	2	[34,35]
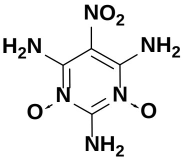	1.95	284	9169	34.3	>60	>360	[36]
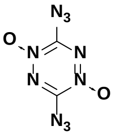	1.9	140	10030	45.8	1.5	10	[37]
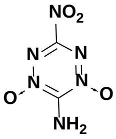	1.92	110	9316	39.4	3	10	[38]
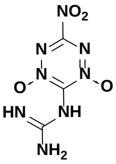	1.91	134	9157	37.5	20	240	[38]
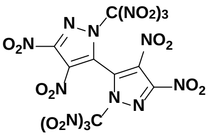	2.02	125	9320	40	9	215	[39]

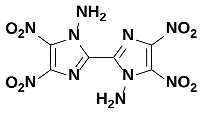	1.93	217	9012	36.6	15	160	[40,41]
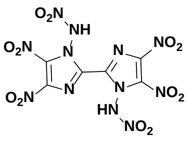	1.94	116	9350	40.1	3	20	[40,42]
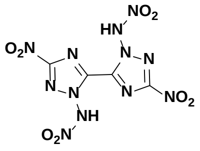	1.88	121	9243	38.2	3	40	[42]
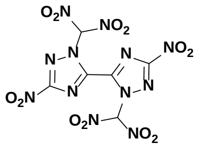	1.95	166	9394	39.7	4	120	[43]
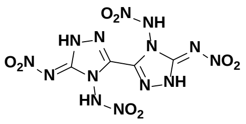	1.91	137	9421	40.3	5	60	[44]
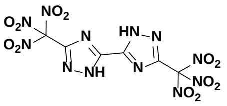	1.89	148	9073	36.2	22.5	252	[15]
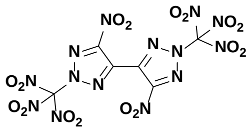	1.92	195	9070	36	5	126	[39,44]
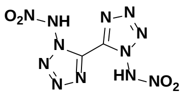	1.93	107	10142	45.6	<1	<5	[45,46]
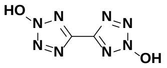	1.95	165	9364	40.9	3	5	[47]
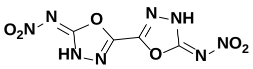	1.99	210	9481	41.9	5	60	[48]

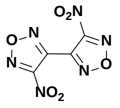	1.85	85	9227	35.6	--	--	[49,50]
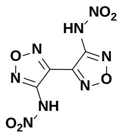	1.94	80	9086	40.3	1.5	48	[51]
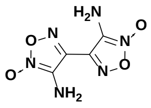	1.86	160	9007	35.4	6	>360	[52]
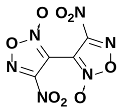	1.97	140	9525	41.2	3.9119	26119	[52]
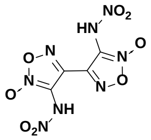	1.91	75	9381	40.8	3	20	[49]
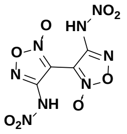	1.94	91	9472	41.7	2	20	[49]
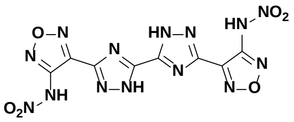	1.94	112	9083	36.3	15.5	240	[49]
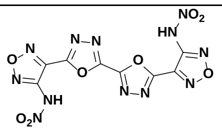	1.92	90	9058	36.2	6.9	120	[53]
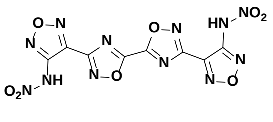	1.92	101	9185	37.5	16	240	[54]
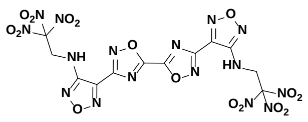	1.9	188	9062	37.2	10.5	240	[54]

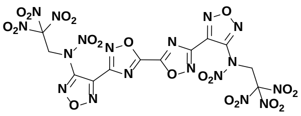	1.93	152	9550	41.9	4.5	120	54
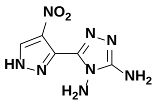	1.87	226	9075	30.7	>80	>360	55
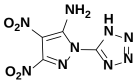	1.85	222	9261	38.3	25	168	56
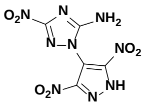	1.84	270	9167	37.8	9	240	57
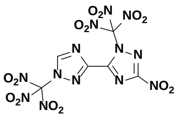	1.92	152	9107	36.2	5	120	57
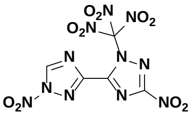	1.9	136	9254	37.7	11	250	57
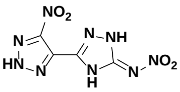	1.88	168	9067	36.2	16	180	55
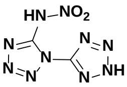	1.77	122	9099	33.6	1	8	58
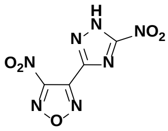	1.86	191	9152	37.1	23	300	59
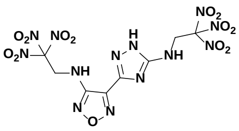	1.91	168	9087	38.1	8	220	59

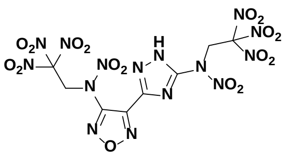	1.93	92	9355	40.1	3	120	[59]
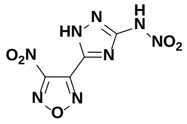	1.85	163	9025	36	20	280	[60]
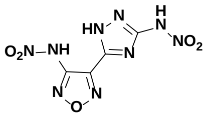	1.92	129	9258	39	18	250	[60]
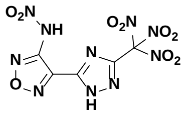	1.89	67	9278	39	4.4	60	[61]
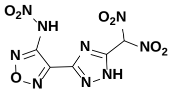	1.85	91	9005	36.3	8.7	100	[61]
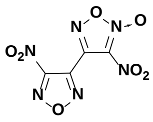	1.92	146	9180	38.8	2.6	67	[62]
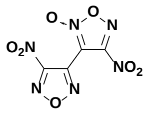	1.93	140	9230	39.4	2.8	82	[62]
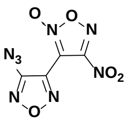	1.83	113	9280	38.1	2	32	[63]
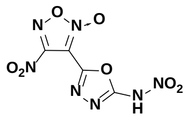	1.88	86	9258	37.5	8	108	[64]
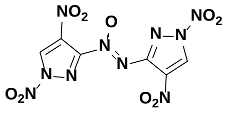	1.83	161	9228	38.7	7	120	[65]

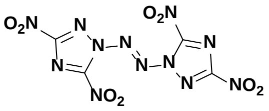	1.93	262	9490	42.4	10	160	66
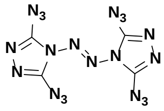	1.76	136	9370	38.4	<3	--	67
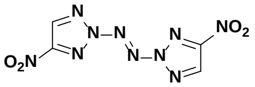	1.84	227	9068	35.4	4.0-4.5	36-40	68
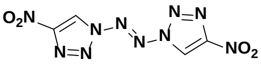	1.82	160	9014	35.4	<1	<5	68
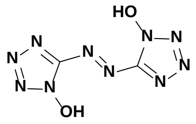	1.9	170	9548	42.4	<1	<5	69
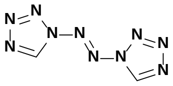	1.77	80	9371	--	<<1	<<5	70
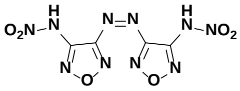	1.89	100	9517	41.1	2	10	71
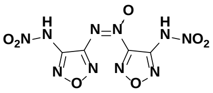	1.96	90	9746	44.1	2	10	72
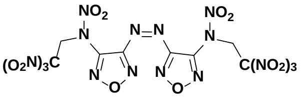	1.87	159	9486	40.8	3.5	70	33
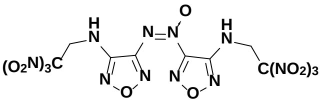	1.84	190	9041	36.7	7.9	130	73
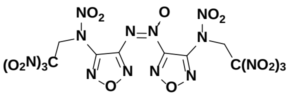	1.92	145	9458	41.2	3.2	80	73

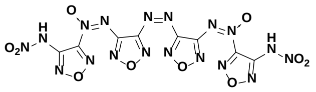	1.88	120	9541	40.5	2	10	74
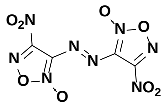	1.98	124	9778	50.2	1	<5	75
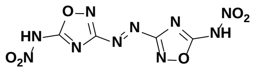	1.9	140	9190	37.5	2	10	76
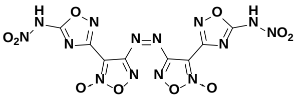	1.92	126	9505	41.3	4	80	77
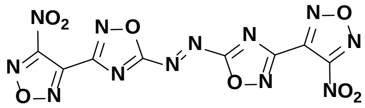	1.92	256	9240	37.5	18	220	78
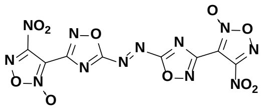	1.92	180	9666	42.8	12	180	79
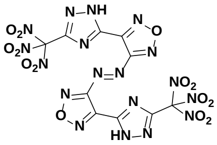	1.9	144	9354	39.2	4.9	160	80
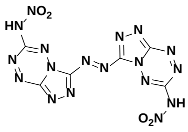	1.85	170	9500	39.8	14	<5	81
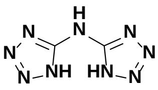	1.86	250	9120	34.3	>30	>360	82
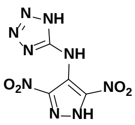	1.86	279	9289	38.6	35	240	83

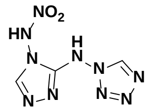	1.78	177	9141	33.6	18	180	84
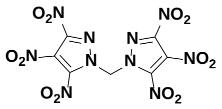	1.93	205	9304	39.1	4	144	85
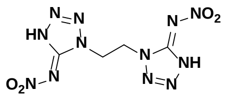	1.86	194	9329	38.2	10	--	86
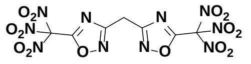	1.91	117	9053	37.4	12.5	72	87
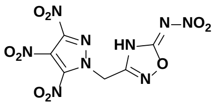	1.84	170	9014	36.7	1.5	80	88
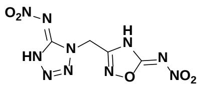	1.88	175	9297	38.3	12	140	89
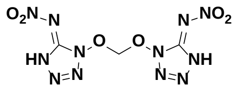	1.95	157	9867	46.7	1	--	90
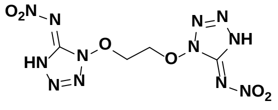	1.81	134	9200	38.4	1.5	--	90
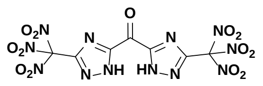	1.93	150	9275	39.3	13	240	91
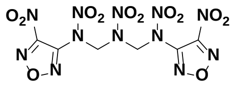	1.9	150	9334	39.3	2	324	34
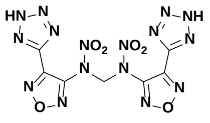	1.83	154	9043	35.6	16	180	92

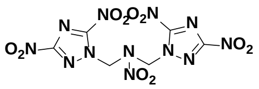	1.9	209	9089	37.3	<1	168	93
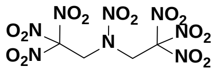	1.96	98	9120	37	4	ND	39,94,95
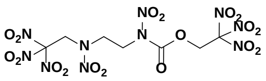	1.89	157	9083	36.5	10	216	96
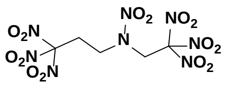	1.89	153	9119	26.4	7	2	97
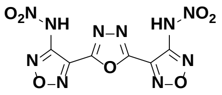	1.91	67	9211	38	--	--	53
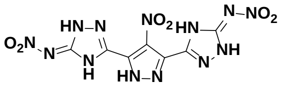	1.92	134	9008	35.9	20	270	98
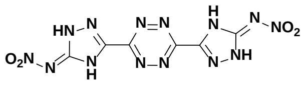	1.88	162	9100	31.7	20	270	99
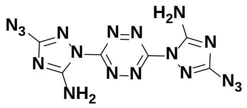	1.91	186	9017	32.8	6.2	>360	100
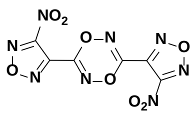	1.87	148	9040	37.7	2.2	116	101
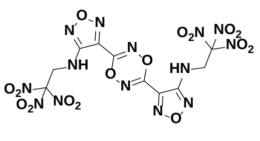	1.94	203	9156	38.9	16.3	240	102
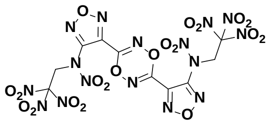	1.95	119	9600	42.8	3.5	100	102

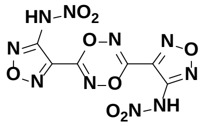	1.93	106	9109	42.8	4.5	100	[102]
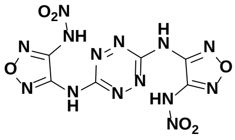	1.85	149	9087	36.2	16	160	[103]
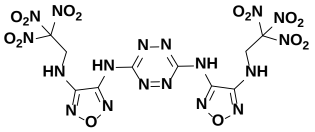	1.86	204	9042	37	21	360	[103]
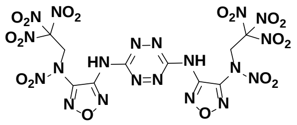	1.92	144	9548	42.1	10	240	[103]
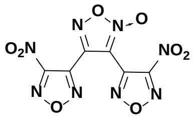	1.87	231	9043	35.5	14	360	[104]
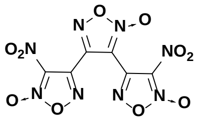	1.98	131	9867	45	3	35	[105]
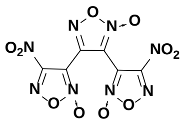	1.91	147	9503	40.8	3	40	[105,106]
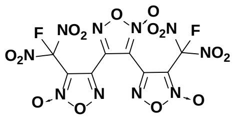	2	116	9509	42.6	5	70	[107]
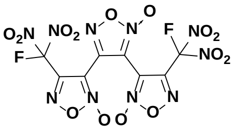	1.91	136	9196	38.8	6.5	110	[107]

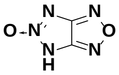	1.93	89	9250	43.1	3.2	31	108
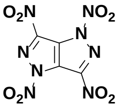	1.95	145	9460	40.9	3	20	109
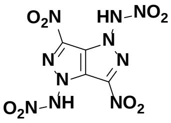	1.93	128	9507	41.8	2	20	109
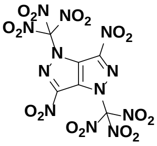	1.99	160	9182	37.8	7	40	110
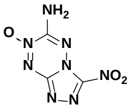	1.86	220	9384	39.1	25	240	111
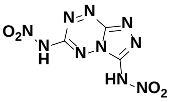	1.91	138	9301	38.3	3	>5	81
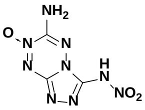	1.88	182	9047	35.1	10	>160	112
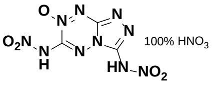	1.93	114	9503	41	1	5	113
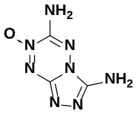	1.82	196	9008	34.7	>40	>360	111
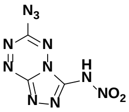	1.85	150	9236	36.3	1	>40	114
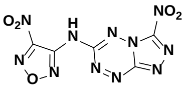	1.86	184	9182	36.7	>40	--	115

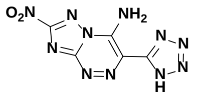	1.87	181	9010	32.8	35	300	[116]
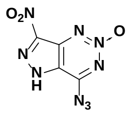	1.86	160	9111	35.1	60	1320	[117]
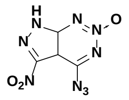	1.85	203	9120	35.1	18	325	[118]
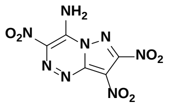	1.95	246	8998	36	14.3	324–360	[119]
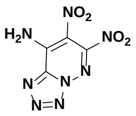	1.89	202	9021	34.8	18	112	[120]
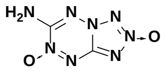	1.93	150	9600	41.3	6	109	[37]
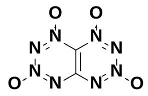	1.98	183	9710	43.2	--	--	[121]
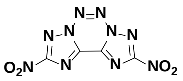	1.91	138	9400	38	5.3	92	[122]
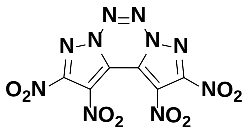	1.96	233	9631	44	10	240	[123]
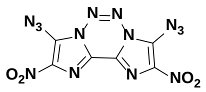	1.89	111	9256	36.7	2	20	[124]
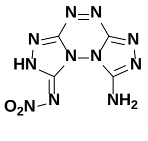	1.88	166	9073	34	>40	>360	[125]

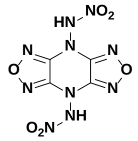	1.8	74	9153	36.5	1	24	126
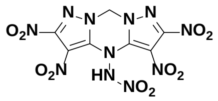	1.94	117	9226	38.8	3	20	127
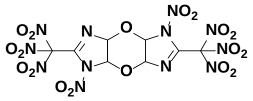	1.91	127	9325	40	--	--	128
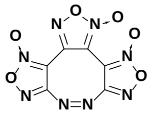	1.9	161	9417	39.6	19	80	106
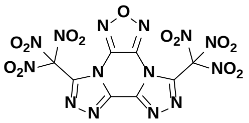	1.89	128	9010	33	11	130	129

4 Supplementary References

1. Guillard, J., Goujon, F., Badol, P. & Poullain, D. New synthetic route to diaminonitropyrazoles as precursors of energetic materials. *Tetrahedron Letters* **44**, 5943–5945 (2003).
2. St John, P. C., Guan, Y., Kim, Y., Etz, B. D., Kim, S., & Paton, R. S. Quantum chemical calculations for over 200,000 organic radical species and 40,000 associated closed-shell molecules. *Sci Data* **7**, 244 (2020).
3. Westwell, M. S., Searle, M. S., Wales, D. J. & Williams, D. H. Empirical Correlations between Thermodynamic Properties and Intermolecular Forces. *J. Am. Chem. Soc.* **117**, 5013–5015 (1995).
4. Lipparini, F., Egidi, F., Goings, J., Peng, B., Petrone, A., Henderson, T. & Fox, D. J. Gaussian 09, revision E. 01. Gaussian Inc.: Wallingford, CT (2013).
5. Lu, T. & Chen, F. Multiwfn: a multifunctional wavefunction analyzer. *Journal of computational chemistry* **33**, 580–592 (2012).
6. Humphrey, W., Dalke, A. & Schulten, K. VMD: visual molecular dynamics. *Journal of molecular graphics* **14**, 33–38 (1996).
7. Hervé, G., Roussel, C. & Graindorge, H. Selective Preparation of 3,4,5-Trinitro-1*H*-Pyrazole: A Stable All-Carbon-Nitrated Arene. *Angew. Chem. Int. Ed.* **49**, 3177–3181 (2010).
8. Du, Y., Zhang, J., Peng, P., Su, H., Li, S., & Pang, S. P. Synthesis and characterization of three pyrazolate inner diazonium salts: green, powerful and stable primary explosives. *New J. Chem.* **41**, 9244–9249 (2017).
9. Yin, P., Parrish, D. A. & Shreeve, J. M. Energetic Multifunctionalized Nitraminopyrazoles and Their Ionic Derivatives: Ternary Hydrogen-Bond Induced High Energy Density Materials. *J. Am. Chem. Soc.* **137**, 4778–4786 (2015).

10. Dalinger, I. L., Vatsadse, I. A., Shkineva, T. K., Popova, G. P., Ugrak, B. I., & Shevelev, S. A. Nitropyrazoles. *Russ Chem Bull* **59**, 1631–1638 (2010).
11. Yin, P., Zhang, J., He, C., Parrish, D. A. & Shreeve, J. M. Polynitro-substituted pyrazoles and triazoles as potential energetic materials and oxidizers. *J. Mater. Chem. A* **2**, 3200 (2014).
12. Xiong, H., Yang, H. & Cheng, G. 3-Trinitromethyl-4-nitro-5-nitramine-1*H*-pyrazole: a high energy density oxidizer. *New J. Chem.* **43**, 13827–13831 (2019).
13. Zhao, X. X., Li, S. H., Wang, Y., Li, Y. C., Zhao, F. Q., & Pang, S. P. Design and synthesis of energetic materials towards high density and positive oxygen balance by N-dinitromethyl functionalization of nitroazoles. *J. Mater. Chem. A* **4**, 5495–5504 (2016).
14. Liu, T., Qi, X., Wang, K., Zhang, J., Zhang, W., & Zhang, Q. Green primary energetic materials based on N-(3-nitro-1-(trinitromethyl)-1H-1,2,4-triazol-5-yl)nitramide. *New J. Chem.* **41**, 9070–9076 (2017).
15. Ma, Q., Gu, H., Huang, J., Nie, F., Fan, G., Liao, L., & Yang, W. Formation of trinitromethyl functionalized 1,2,4-triazole-based energetic ionic salts and a zwitterionic salt directed by an intermolecular and intramolecular metathesis strategy. *New J. Chem.* **42**, 2376–2380 (2018).
16. Hermann, T. S., Klapötke, T. M., Krumm, B. & Stierstorfer, J. The energetic 3-trinitromethyl-5-nitramino-1H-1,2,4-triazole and nitrogen-rich salts. *New J. Chem.* **41**, 3068–3072 (2017).
17. Thottempudi, V. & Shreeve, J. M. Synthesis and Promising Properties of a New Family of High-Density Energetic Salts of 5-Nitro-3-trinitromethyl-1*H*-1,2,4-triazole and 5,5′-Bis(trinitromethyl)-3,3′-azo-1*H*-1,2,4-triazole. *J. Am. Chem. Soc.* **133**, 19982–19992 (2011).
18. Thottempudi, V., Gao, H. & Shreeve, J. M. Trinitromethyl-Substituted 5-Nitro- or 3-Azo-1,2,4-triazoles: Synthesis, Characterization, and Energetic Properties. *J. Am. Chem. Soc.* **133**, 6464–6471 (2011).
19. Dharavath, S., Zhang, J., Imler, G. H., Parrish, D. A. & Shreeve, J. M. 5-(Dinitromethyl)-3-(trinitromethyl)-1,2,4-triazole and its derivatives: a new application of oxidative nitration towards *gem*-trinitro-based energetic materials. *J. Mater. Chem. A* **5**, 4785–4790 (2017).
20. Fischer, N., Klapötke, T. M. & Stierstorfer, J. New Nitriminotetrazoles - Synthesis, Structures and Characterization. *Z. anorg. allg. Chem.* **635**, 271–281 (2009).
21. Klapötke, T. M. & Stierstorfer, J. Nitration Products of 5-Amino-1H-tetrazole and Methyl-5-amino-1H-tetrazoles – Structures and Properties of Promising Energetic Materials. *HCA* **90**, 2132–2150 (2007).
22. Klapötke, T. M., Sabaté, C. M. & Stierstorfer, J. Neutral 5-nitrotetrazoles: easy initiation with low pollution. *New J. Chem.* **33**, 136–147 (2009).
23. Klapötke, T. M., Mayer, P., Miró Sabaté, C., Welch, J. M. & Wiegand, N. Simple, Nitrogen-Rich, Energetic Salts of 5-Nitrotetrazole. *Inorg. Chem.* **47**, 6014–6027 (2008).
24. Klapötke, T. M., Piercey, D. G. & Stierstorfer, J. Amination of energetic anions: high-performing energetic materials. *Dalton Trans.* **41**, 9451 (2012).
25. Göbel, M., Karaghiosoff, K., Klapötke, T. M., Piercey, D. G. & Stierstorfer, J. Nitrotetrazolate-2*N*-oxides and the Strategy of *N*-Oxide Introduction. *J. Am. Chem. Soc.* **132**, 17216–17226 (2010).
26. Fischer, D., Klapötke, T. M. & Stierstorfer, J. 1,5-Di(nitramino)tetrazole: High Sensitivity and Superior Explosive Performance. *Angew. Chem. Int. Ed.* **54**, 10299–10302 (2015).
27. Klapötke, T. M., Krumm, B., Martin, F. A. & Stierstorfer, J. New Azidotetrazoles: Structurally Interesting and Extremely Sensitive. *Chem. Asian J.* **7**, 214–224 (2012).
28. Haiges, R. & Christe, K. O. Energetic High-Nitrogen Compounds: 5-(Trinitromethyl)-2*H*-tetrazole and -tetrazolates, Preparation, Characterization, and Conversion into 5-(Dinitromethyl)tetrazoles. *Inorg. Chem.* **52**, 7249–7260 (2013).
29. Klapötke, T. M. & Steemann, F. X. Dinitromethyltetrazole and its Salts - A Comprehensive Study. *Propellants, Explosives, Pyrotechnics* **35**, 114–129 (2010).

30. Yu, Q., Imler, G. H., Parrish, D. A. & Shreeve, J. M. Challenging the Limits of Nitro Groups Associated with a Tetrazole Ring. *Org. Lett.* **21**, 4684–4688 (2019).
31. Fu, Z., Su, R., Wang, Y., Wang, Y. F., Zeng, W., Xiao, N., Wu, Y., Zhou, Z. M., Chen, J. & Chen, F. X. Synthesis and Characterization of Energetic 3-Nitro-1,2,4-oxadiazoles. *Chem. Eur. J.* **18**, 1886–1889 (2012).
32. Tang, Y., Zhang, J., Mitchell, L. A., Parrish, D. A. & Shreeve, J. M. Taming of 3,4-Di(nitramino)furazan. *J. Am. Chem. Soc.* **137**, 15984–15987 (2015).
33. Yu, Q., Wang, Z., Wu, B., Yang, H., Ju, X., Lu, C., & Cheng, G. A study of N-trinitroethyl-substituted aminofurazans: high detonation performance energetic compounds with good oxygen balance. *J. Mater. Chem. A* **3**, 8156–8164 (2015).
34. Klapötke, T. M. & Pflüger, C. 3-Nitramino-4-nitrofurazan: Enhancing the Stability and Energetic Properties by Introduction of Alkylnitramines. *Z. Anorg. Allg. Chem.* **643**, 619–624 (2017).
35. Klapötke, T., Schmid, P. & Stierstorfer, J. Crystal Structures of Furazanes. *Crystals* **5**, 418–432 (2015).
36. Wang, Y., Liu, Y., Song, S., Yang, Z., Qi, X., Wang, K., Liu, Y., Zhang, Q. H. & Tian, Y. Accelerating the discovery of insensitive high-energy-density materials by a materials genome approach. *Nature Communications* **9**, 2444 (2018).
37. Chavez, D. E., Parrish, D. A., Mitchell, L. & Imler, G. H. Azido and Tetrazolo 1,2,4,5-Tetrazine N-Oxides. *Angew. Chem. Int. Ed.* **56**, 3575–3578 (2017).
38. Wei, H., Gao, H. & Shreeve, J. M. *N*-Oxide 1,2,4,5-Tetrazine-Based High-Performance Energetic Materials. *Chem. Eur. J.* **20**, 16943–16952 (2014).
39. Dalinger, I. L., Suponitsky, K. Yu., Shkineva, T. K., Lempert, D. B. & Sheremetev, A. B. Bipyrazole bearing ten nitro groups – a novel highly dense oxidizer for forward-looking rocket propulsions. *J. Mater. Chem. A* **6**, 14780–14786 (2018).
40. Yin, P., He, C. & Shreeve, J. M. Fully C/N-Polynitro-Functionalized 2,2′-Biimidazole Derivatives as Nitrogen- and Oxygen-Rich Energetic Salts. *Chem. Eur. J.* **22**, 2108–2113 (2016).
41. Chavez, D., Breiner, M. & Parrish, D. Nucleophilic Reactions of The Bis Ammonium Salt of 4,4′,5,5′-Tetranitro-2,2′-biimidazole. *Synlett* **24**, 519–521 (2013).
42. Yin, P. & Shreeve, J. M. From *N*-Nitro to *N*-Nitroamino: Preparation of High-Performance Energetic Materials by Introducing Nitrogen-Containing Ions. *Angew. Chem. Int. Ed.* **54**, 14513–14517 (2015).
43. Huang, S., Tian, J., Qi, X., Wang, K. & Zhang, Q. Synthesis of *gem* -Dinitromethylated and Fluorodinitromethylated Derivatives of 5,5′-Dinitro-bis-1,2,4-triazole as Promising High-Energy-Density Materials. *Chem. Eur. J.* **23**, 12787–12794 (2017).
44. Semenov, V. V., Shevelev, S. A., Bruskin, A. B., Shakhnes, A. Kh. & Kuz'min, V. S. Synthesis of 5,5'-dinitro-2,2'-bis(polynitromethyl)-bi(1,2,3(4)-triazoles), hydrogen-free oxidizers. *Chem Heterocycl Comp* **53**, 728–732 (2017).
45. Fischer, D., Klapötke, T. M., Stierstorfer, J. & Szimhardt, N. 1,1′-Nitramino-5,5′-bitetrazoles. *Chem. Eur. J.* **22**, 4966–4970 (2016).
46. Fischer, D., Klapötke, T. M. & Stierstorfer, J. Potassium 1,1′-Dinitramino-5,5′-bistetrazolate: A Primary Explosive with Fast Detonation and High Initiation Power. *Angew. Chem. Int. Ed.* **53**, 8172–8175 (2014).
47. Fischer, N., Gao, L., Klapötke, T. M. & Stierstorfer, J. Energetic salts of 5,5′-bis(tetrazole-2-oxide) in a comparison to 5,5′-bis(tetrazole-1-oxide) derivatives. *Polyhedron* **51**, 201–210 (2013).
48. Zhang, W., Zhang, J., Deng, M., Qi, X., Nie, F., & Zhang, Q. A promising high-energy-density material. *Nat Commun* **8**, 181 (2017).
49. He, C., Tang, Y., Mitchell, L. A., Parrish, D. A. & Shreeve, J. M. *N*-Oxides light up energetic performances: synthesis and characterization of dinitraminobisfuroxans and their salts. *J. Mater. Chem. A* **4**, 8969–8973 (2016).
50. Coburn, M. D. Picrylamino-substituted heterocycles. II. Furazans. *Journal of Heterocyclic Chemistry* **5**, 83–87 (1968).

51. Fischer, D., Klapötke, T. M., Reymann, M. & Stierstorfer, J. Dense Energetic Nitraminofurazanes. *Chem. Eur. J.* **20**, 6401–6411 (2014).
52. Fischer, D., Klapötke, T. M. & Stierstorfer, J. Synthesis and Characterization of Diaminobisfuroxane. *Eur. J. Inorg. Chem.* **2014**, 5808–5811 (2014).
53. Ma, J., Chinnam, A. K., Cheng, G., Yang, H., Zhang, J., & Shreeve, J. N. M. 1,3,4-Oxadiazole Bridges: A Strategy to Improve Energetics at the Molecular Level. *Angew Chem Int Ed* **60**, 5497–5504 (2021).
54. Wang, B., Xiong, H., Cheng, G. & Yang, H. Incorporating Energetic Moieties into Four Oxadiazole Ring Systems for the Generation of High-Performance Energetic Materials. *ChemPlusChem* **83**, 439–447 (2018).
55. Ma, Q., Zhang, G., Li, J., Zhang, Z., Lu, H., Liao, L., Fan, G. J. & Nie, F. Pyrazol-triazole energetic hybrid with high thermal stability and decreased sensitivity: facile synthesis, characterization and promising performance. *Chemical Engineering Journal* **379**, 122331 (2020).
56. Fu, W., Zhao, B., Zhang, M., Li, C., Gao, H., Zhang, J., & Zhou, Z. 3,4-Dinitro-1-(1H-tetrazol-5-yl)-1H-pyrazol-5-amine (HANTP) and its salts: primary and secondary explosives. *J. Mater. Chem. A* **5**, 5044–5054 (2017).
57. Li, C., Zhang, M., Chen, Q., Li, Y., Gao, H., Fu, W., & Zhou, Z. 1-(3,5-Dinitro-1H-pyrazol-4-yl)-3-nitro-1H-1,2,4-triazol-5-amine (HCPT) and its energetic salts: highly thermally stable energetic materials with high-performance. *Dalton Trans.* **45**, 17956–17965 (2016).
58. Wang, B., Qi, X., Zhang, W., Wang, K., Li, W., & Zhang, Q. Synthesis of 1-(2H-tetrazol-5-yl)-5-nitraminotetrazole and its derivatives from 5-aminotetrazole and cyanogen azide: a promising strategy towards the development of C–N linked bistetrazolate energetic materials. *J. Mater. Chem. A* **5**, 20867–20873 (2017).
59. Xu, Z., Cheng, G., Yang, H., Zhang, J. & Shreeve, J. M. Synthesis and Characterization of 4-(1,2,4-Triazole-5-yl)furazan Derivatives as High-Performance Insensitive Energetic Materials. *Chem. Eur. J.* **24**, 10488–10497 (2018).
60. Xu, Z., Cheng, G., Yang, H., Ju, X., Yin, P., Zhang, J., & Shreeve, J. N. M. A Facile and Versatile Synthesis of Energetic Furazan-Functionalized 5-Nitroimino-1,2,4-Triazoles. *Angew. Chem. Int. Ed.* **56**, 5877–5881 (2017).
61. Ma, J., Tang, J., Yang, H., Yi, Z., Wu, G., Zhu, S., Zhang, W., Li, Y & Cheng, G. Polynitro-Functionalized Triazolylfurazanate Triaminoguanidine: Novel Green Primary Explosive with Insensitive Nature. *ACS Appl. Mater. Interfaces* **11**, 26053–26059 (2019).
62. Fershtat, L. L., Ovchinnikov, I. V., Epishina, M. A., Romanova, A. A., Lempert, D. B., Muravyev, N. V., & Makhova, N. N. Assembly of Nitrofurazan and Nitrofuroxan Frameworks for High-Performance Energetic Materials. *ChemPlusChem* **82**, 1315–1319 (2017).
63. Larin, A. A., Shaferov, A. V., Epishina, M. A., Melnikov, I. N., Muravyev, N. V., Ananyev, I. V. & Makhova, N. N. Pushing the Energy-Sensitivity Balance with High-Performance Bifuroxans. *ACS Appl. Energy Mater.* **3**, 7764–7771 (2020).
64. Qian, L., Yang, H., Xiong, H., Gu, H., Tang, J., Xue, Y., & Cheng, G. Low sensitive energetic material based on the combination of furoxan and 1,3,4-oxadiazole structures. *Energetic Materials Frontiers* **1**, 74–82 (2020).
65. Yang, P., Yang, H., Zhao, Y., Tang, J. & Cheng, G. Novel polynitro azoxypyrazole-based energetic materials with high performance. *Dalton Trans.* **50**, 16499–16503 (2021).
66. Li, Y., Wang, B., Chang, P., Hu, J., Chen, T., Wang, Y., & Wang, B. Novel catenated N_6energetic compounds based on substituted 1,2,4-triazoles: synthesis, structures and properties. *RSC Adv.* **8**, 13755–13763 (2018).
67. Qi, C., Li, S. H., Li, Y. C., Wang, Y., Zhao, X. X., & Pang, S. P. Synthesis and Promising Properties of a New Family of High-Nitrogen Compounds: Polyazido- and Polyamino-Substituted*N*,*N′*-Azo-1,2,4-triazoles. *Chem. Eur. J.* **18**, 16562–16570 (2012).
68. Wozniak, D. R., Salfer, B., Zeller, M., Byrd, E. F. C. & Piercey, D. G. Tailoring Energetic Sensitivity and Classification through Regioisomerism. *Org. Lett.* **22**, 9114–9117 (2020).
69. Fischer, D., Klapötke, T. M., Piercey, D. G. & Stierstorfer, J. Synthesis of 5-Aminotetrazole-1 *N*-oxide and Its Azo Derivative: A Key Step in the Development of New Energetic Materials. *Chem. Eur. J.* **19**, 4602–4613 (2013).

70. Klapötke, T. M. & Piercey, D. G. 1,1′-Azobis(tetrazole): A Highly Energetic Nitrogen-Rich Compound with a N_{10}Chain. *Inorg. Chem.* **50**, 2732–2734 (2011).
71. Zhang, J. & Shreeve, J. M. Nitroaminofurazans with Azo and Azoxy Linkages: A Comparative Study of Structural, Electronic, Physicochemical, and Energetic Properties. *J. Phys. Chem. C* **119**, 12887–12895 (2015).
72. Zhang, J. & Shreeve, J. M. 3,3′-Dinitroamino-4,4′-azoxyfurazan and Its Derivatives: An Assembly of Diverse N–O Building Blocks for High-Performance Energetic Materials. *J. Am. Chem. Soc.* **136**, 4437–4445 (2014).
73. Yu, Q., Wang, Z., Yang, H., Wu, B., Lin, Q., Ju, X., Lu, C. & Cheng, G. N-Trinitroethyl-substituted azoxyfurazan: high detonation performance energetic materials. *RSC Adv.* **5**, 27305–27312 (2015).
74. Liu, Y., Zhang, J., Wang, K., Li, J., Zhang, Q., & Shreeve, J. N. M. Bis(4-nitraminofurazanyl-3-azoxy)azofurazan and Derivatives: 1,2,5-Oxadiazole Structures and High-Performance Energetic Materials. *Angew. Chem. Int. Ed.* **55**, 11548–11551 (2016).
75. Guo, T., Liu, M., Huang, X. C., Wang, Z. J., Qiu, S. J., Ge, Z. X., & Meng, Z. H. Efficient preparation and comprehensive properties of thermal decomposition and detonation for 4,4′-dinitro-3,3′-azofuroxan. *Journal of Analytical and Applied Pyrolysis* **128**, 451–458 (2017).
76. Tang, Y., Gao, H., Mitchell, L. A., Parrish, D. A. & Shreeve, J. M. Syntheses and Promising Properties of Dense Energetic 5,5′-Dinitramino-3,3′-azo-1,2,4-oxadiazole and Its Salts. *Angew. Chem. Int. Ed.* **55**, 3200–3203 (2016).
77. Xiong, H., Yang, H., Lei, C., Yang, P., Hu, W., & Cheng, G. Combinations of furoxan and 1,2,4-oxadiazole for the generation of high performance energetic materials. *Dalton Trans.* **48**, 14705–14711 (2019).
78. Wang, Q., Shao, Y. & Lu, M. $C_8N_{12}O_8$: A Promising Insensitive High-Energy-Density Material. *Crystal Growth & Design* **18**, 6150–6154 (2018).
79. Xiong, H., Cheng, G., Zhang, Z. & Yang, H. $C_8N_{12}O_{10}$: a promising energetic compound with excellent detonation performance and desirable sensitivity. *New J. Chem.* **43**, 7784–7789 (2019).
80. Ma, J., Zhang, G., Gu, H., Tang, J., Wang, B., Yang, H., & Cheng, G. Polynitro-1,2,4-triazole-functionalized azo-furazans as high-performance and insensitive energetic materials. *New J. Chem.* **43**, 8370–8375 (2019).
81. Hu, L., Yin, P., Zhao, G., He, C., Imler, G. H., Parrish, D. A., ... & Shreeve, J. N. M. Conjugated Energetic Salts Based on Fused Rings: Insensitive and Highly Dense Materials. *J. Am. Chem. Soc.* **140**, 15001–15007 (2018).
82. Klapötke, T. M., Mayer, P., Stierstorfer, J. & Weigand, J. J. Bistetrazolylamines—synthesis and characterization. *J. Mater. Chem.* **18**, 5248 (2008).
83. Zhang, M., Gao, H., Li, C., Fu, W., Tang, L., & Zhou, Z. Towards improved explosives with a high performance: N-(3,5-dinitro-1H-pyrazol-4-yl)-1H-tetrazol-5-amine and its salts. *J. Mater. Chem. A* **5**, 1769–1777 (2017).
84. Tang, J., Yang, P., Yang, H., Xiong, H., Hu, W., & Cheng, G. A simple and efficient method to synthesize high-nitrogen compounds: Incorporation of tetrazole derivatives with N5 chains. *Chemical Engineering Journal* **386**, 124027 (2020).
85. Fischer, D., Gottfried, J. L., Klapötke, T. M., Karaghiosoff, K., Stierstorfer, J., & Witkowski, T. G. Synthesis and Investigation of Advanced Energetic Materials Based on Bispyrazolylmethanes. *Angew. Chem. Int. Ed.* **55**, 16132–16135 (2016).
86. Joo, Y.-H. & Shreeve, J. M. Energetic Mono-, Di-, and Trisubstituted Nitroiminotetrazoles. *Angew. Chem. Int. Ed.* **48**, 564–567 (2009).
87. Lu, T., Wang, C., Wang, G., Wang, S., Song, J., Yin, H., Fan, G. J. & Chen, F. X. 1,2,4-Oxadiazole-derived polynitro energetic compounds with sensitivity reduced by a methylene bridge. *New J. Chem.* **43**, 13330–13333 (2019).
88. Yang, F., Zhang, P., Zhou, X., Lin, Q., Wang, P., & Lu, M. Combination of Polynitropyrazole and 5-Amino-1,2,4-oxadiazole Derivatives: An Approach to High Performance Energetic Materials. *Crystal Growth & Design* **20**, 3737–3746 (2020).
89. Wang, Y., Ye, J., Yang, N., Ma, H., Zhang, Y., & Guo, Z. Strong intermolecular interaction induced methylene-bridged asymmetric heterocyclic explosives. *CrystEngComm* **23**, 7635–7642 (2021).

90. Joo, Y.-H. & Shreeve, J. M. High-Density Energetic Mono- or Bis(Oxy)-5-Nitroiminotetrazoles. *Angewandte Chemie International Edition* **49**, 7320–7323 (2010).
91. Chinnam, A. K., Singh, J., Staples, R. J. & Shreeve, J. M. Bis(3-(trinitromethyl)-1H-1,2,4-triazol-5-yl)methanone: A mildly acidic high-performing energetic material. *Chemical Engineering Journal* **433**, 133520 (2022).
92. Sun, Q., Shen, C., Li, X., Lin, Q. & Lu, M. 3D-Cube Layer Stacking: A Promising Strategy for High-Performance Insensitive Energetic Materials. *Crystal Growth & Design* **17**, 6105–6110 (2017).
93. Klapötke, T. M., Penger, A., Pflüger, C., Stierstorfer, J. & Sućeska, M. Advanced Open-Chain Nitramines as Energetic Materials: Heterocyclic-Substituted 1,3-Dichloro-2-nitrazapropane. *Eur. J. Inorg. Chem.* **2013**, 4667–4678 (2013).
94. Fedorov, B. S. Novel transformations of nitraminodiol diacetates. *Russ Chem Bull* **45**, 722–723 (1996).
95. Klapötke, T. M., Krumm, B., Scherr, M., Steemann, F. X., Banert, K., & Joo, Y. H. Experimental and Theoretical Studies on Some Energetic Functionalized Trimethylamine Derivatives. *Chem. Eur. J.* **15**, 11341–11345 (2009).
96. Klapötke, T. M., Krumm, B. & Scharf, R. From Amino Acids to High-Energy Dense Oxidizers: Polynitro Materials Derived from β-Alanine and<scp>L</scp>-Aspartic Acid. *Z. anorg. allg. Chem.* **642**, 887–895 (2016).
97. Axthammer, Q. J., Krumm, B., Klapötke, T. M. & Scharf, R. A Study of the 3,3,3-Trinitropropyl Unit as a Potential Energetic Building Block. *Chem. Eur. J.* **21**, 16229–16239 (2015).
98. Xu, M., Cheng, G., Xiong, H., Wang, B., Ju, X., & Yang, H. Synthesis of high-performance insensitive energetic materials based on nitropyrazole and 1,2,4-triazole. *New J. Chem.* **43**, 11157–11163 (2019).
99. Zhang, S., Cheng, G. & Yang, H. Studies on the synthesis and properties of nitramino compounds based on tetrazine backbones. *Dalton Trans.* **49**, 5590–5596 (2020).
100. Shlomovich, A., Pechersky, T., Cohen, A., Yan, Q. L., Kosa, M., Petrutik, N. & Gozin, M. Energetic isomers of 1,2,4,5-tetrazine-bis-1,2,4-triazoles with low toxicity. *Dalton Trans.* **46**, 5994–6002 (2017).
101. Leonard, P., Pollard, C., Chavez, D., Rice, B. & Parrish, D. 3,6-Bis(4-nitro-1,2,5-oxadiazol-3-yl)-1,4,2,5-dioxadiazene (BNDD): A Powerful Sensitive Explosive. *Synlett* **2011**, 2097–2099 (2011).
102. Yu, Q., Cheng, G., Ju, X., Lu, C., Lin, Q., & Yang, H. An interesting 1,4,2,5-dioxadiazine-furazan system: structural modification by incorporating versatile functionalities. *Dalton Trans.* **46**, 14301–14309 (2017).
103. Yu, Q., Yang, H., Imler, G. H., Parrish, D. A., Cheng, G., & Shreeve, J. N. M. Derivatives of 3,6-Bis(3-aminofurazan-4-ylamino)-1,2,4,5-tetrazine: Excellent Energetic Properties with Lower Sensitivities. *ACS Appl. Mater. Interfaces* **12**, 31522–31531 (2020).
104. Yu, Q., Chinnam, A. K., Yin, P., Imler, G. H., Parrish, D. A., & Jean'ne, M. S. Finding furoxan rings. *J. Mater. Chem. A* **8**, 5859–5864 (2020).
105. Zhai, L., Bi, F., Luo, Y., Wang, N., Zhang, J., & Wang, B. New Strategy for Enhancing Energetic Properties by Regulating Trifuroxan Configuration: 3,4-Bis(3-nitrofuroxan-4-yl)furoxan. *Sci Rep* **9**, 4321 (2019).
106. He, C., Gao, H., Imler, G. H., Parrish, D. A. & Shreeve, J. M. Boosting energetic performance by trimerizing furoxan. *J. Mater. Chem. A* **6**, 9391–9396 (2018).
107. Zhai, L., Bi, F., Luo, Y., Sun, L., Huo, H., Zhang, J., Wang, B. Z. & Chen, S. Exploring the highly dense energetic materials via regiochemical modulation: A comparative study of two fluorodinitromethyl-functionalized herringbone trifuroxans. *Chemical Engineering Journal* **391**, 123573 (2020).
108. Voronin, A. A., Fedyanin, I. V., Churakov, A. M., Pivkina, A. N., Muravyev, N. V., Strelenko, Y. A. & Tartakovsky, V. A. 4*H*-[1,2,3]Triazolo[4,5-*c*][1,2,5]oxadiazole 5-oxide and Its Salts: Promising Multipurpose Energetic Materials. *ACS Appl. Energy Mater.* **3**, 9401–9407 (2020).
109. Yin, P., Zhang, J., Mitchell, L. A., Parrish, D. A. & Shreeve, J. M. 3,6-Dinitropyrazolo[4,3-*c*]pyrazole-Based Multipurpose Energetic Materials through Versatile N-Functionalization Strategies. *Angew. Chem. Int. Ed.* **55**, 12895–12897 (2016).
110. Mohammad, K., Thaltiri, V., Kommu, N. & Vargeese, A. A. Octanitropyrazolopyrazole: a gem-trinitromethyl based

green high-density energetic oxidizer. *Chem. Commun.* **56**, 12945–12948 (2020).

111. Hu, L., Yin, P., Imler, G. H., Parrish, D. A., Gao, H., & Jean’ne, M. S. Fused rings with *N*-oxide and –NH_2: good combination for high density and low sensitivity energetic materials. *Chem. Commun.* **55**, 8979–8982 (2019).
112. Hu, L., He, C., Zhao, G., Imler, G. H., Parrish, D. A., & Shreeve, J. N. M. Selecting Suitable Substituents for Energetic Materials Based on a Fused Triazolo-[1,2,4,5]tetrazine Ring. *ACS Appl. Energy Mater.* **3**, 5510–5516 (2020).
113. Hu, L., Gao, H. & Shreeve, J. M. Challenging the limits of nitrogen and oxygen content of fused rings. *J. Mater. Chem. A* **8**, 17411–17414 (2020).
114. Liu, Y., Zhao, G., Tang, Y., Zhang, J., Hu, L., Imler, G. H., Parrish, D. A. & Jean'ne, M. S. Multipurpose [1,2,4]triazolo[4,3-*b*][1,2,4,5] tetrazine-based energetic materials. *J. Mater. Chem. A* **7**, 7875–7884 (2019).
115. Wang, G., Lu, T., Fan, G., Li, C., Yin, H., & Chen, F. X. The Chemistry and Properties of Energetic Materials Bearing [1,2,4]Triazolo[4,3-*b*][1,2,4,5]tetrazine Fused Rings. *Chem. Asian J.* **13**, 3718–3722 (2018).
116. Wang, Q., Shao, Y. & Lu, M. Amino-tetrazole functionalized fused triazolo-triazine and tetrazolo-triazine energetic materials. *Chem. Commun.* **55**, 6062–6065 (2019).
117. Deng, M., Feng, Y., Zhang, W., Qi, X. & Zhang, Q. A green metal-free fused-ring initiating substance. *Nat Commun* **10**, 1339 (2019).
118. Feng, Y., Deng, M., Song, S., Chen, S., Zhang, Q., & Jean'ne, M. S. Construction of an Unusual Two-Dimensional Layered Structure for Fused-Ring Energetic Materials with High Energy and Good Stability. *Engineering* **6**, 1006–1012 (2020).
119. Schulze, M. C., Scott, B. L. & Chavez, D. E. A high density pyrazolo-triazine explosive (PTX). *J. Mater. Chem. A* **3**, 17963–17965 (2015).
120. Chen, S., Liu, Y., Feng, Y., Yang, X. & Zhang, Q. 5,6-Fused bicyclic tetrazolo-pyridazine energetic materials. *Chem. Commun.* **56**, 1493–1496 (2020).
121. Klenov, M. S., Guskov, A. A., Anikin, O. V., Churakov, A. M., Strelenko, Y. A., Fedyanin, I. V. & Tartakovsky, V. A. Synthesis of Tetrazino-tetrazine 1,3,6,8-Tetraoxide (TTTO). *Angew. Chem. Int. Ed.* **55**, 11472–11475 (2016).
122. Chavez, D. E., Bottaro, J. C., Petrie, M. & Parrish, D. A. Synthesis and Thermal Behavior of a Fused, Tricyclic 1,2,3,4-Tetrazine Ring System. *Angew. Chem. Int. Ed.* **54**, 12973–12975 (2015).
123. Tang, Y., Kumar, D. & Shreeve, J. M. Balancing Excellent Performance and High Thermal Stability in a Dinitropyrazole Fused 1,2,3,4-Tetrazine. *J. Am. Chem. Soc.* **139**, 13684–13687 (2017).
124. Tang, Y., He, C., Yin, P., Imler, G. H., Parrish, D. A., & Shreeve, J. N. M. Energetic Functionalized Azido/Nitro Imidazole Fused 1,2,3,4-Tetrazine. *Eur. J. Org. Chem.* **2018**, 2273–2276 (2018).
125. Yu, Q., Singh, J., Staples, R. J. & Shreeve, J. M. Assembling Nitrogen-rich, thermally Stable, and insensitive energetic materials by polycyclization. *Chemical Engineering Journal* **431**, 133235 (2022).
126. Li, W., Wang, K., Qi, X., Jin, Y. & Zhang, Q. Construction of a Thermally Stable and Highly Energetic Metal–Organic Framework as Lead-Free Primary Explosives. *Crystal Growth & Design* **18**, 1896–1902 (2018).
127. Yin, P., Zhang, J., Imler, G. H., Parrish, D. A. & Shreeve, J. M. Polynitro-Functionalized Dipyrazolo-1,3,5-triazinanes: Energetic Polycyclization toward High Density and Excellent Molecular Stability. *Angew. Chem. Int. Ed.* **56**, 8834–8838 (2017).
128. Wu, M., Chen, S., Shu, Q., Li, L. & Jin, S. Synthesis and Characterization of 1,5-Dinitro-2,6-bis(trinitromethyl)-3a,4a,7a,8a-tetrahydro-[1,4]dioxino[2,3-d:5,6-d′]diimidazole (DNTNDI). *Propellants, Explosives, Pyrotechnics* **38**, 658–664 (2013).
129. Sheremetev, A. B., Korolev, V. L., Potemkin, A. A., Aleksandrova, N. S., Palysaeva, N. V., Hoang, T. H., S, V. P. & Suponitsky, K. Y. Oxygen-Rich 1,2,4-Triazolo[3,4-*d*]-1,2,4-triazolo[3,4-*f*]furazano[3,4-*b*]pyrazines as Energetic Materials. *Asian J. Org. Chem.* **5**, 1388–1397 (2016).
